# Supplementary material for: Potent and Selective KDM5 Inhibitor Stops Cellular Demethylation of H3K4me3 at Transcription Start Sites and Proliferation of MM1S Myeloma Cells
Source: Cell Chem Biol. 2017 Mar 16;24(3):371–80. doi: 10.1016/j.chembiol.2017.02.006 (PMC5361737; doi:10.1016/j.chembiol.2017.02.006)
Supplement: Document S1. Supplemental Experimental Procedures, Figures S1–S3, and Tables S1–S5 [file mmc1.pdf]

Cell Chemical Biology, Volume *24*

Supplemental Information

Potent and Selective KDM5 Inhibitor Stops Cellular Demethylation of H3K4me3 at Transcription Start Sites and Proliferation of MM1S Myeloma Cells

Anthony Tumber, Andrea Nuzzi, Edward S. Hookway, Stephanie B. Hatch, Srikannathasan Velupillai, Catrine Johansson, Akane Kawamura, Pavel Savitsky, Clarence Yapp, Aleksandra Szykowska, Na Wu, Chas Bountra, Claire Strain-Damerell, Nicola A. Burgess-Brown, Gian Filippo Ruda, Oleg Fedorov, Shonagh Munro, Katherine S. England, Radoslaw P. Nowak, Christopher J. Schofield, Nicholas B. La Thangue, Charlotte Pawlyn, Faith Davies, Gareth Morgan, Nick Athanasou, Susanne Müller, Udo Oppermann, and Paul E. Brennan

Supplemental Information for

A Selective KDM5 Inhibitor, KDOAM-25, inhibits proliferation of MM1S myeloma cells in vitro.

Table S1 related to Figure 1 2-Oxoglutarate Oxygenase Assay Panel

pIC_{50}*	KDOAM-1	KDOAM-20	KDOAM-21	KDOAM-28	KDOAM-29	KDOAM-25	KDOAM-32
**KDM5**[‡]							
A	*ND*	8.0 ± 0.11 (4)	7.2 ± 0.046 (2)	6.6 ± 0.11 (2)	6.5 ± 0.12 (2)	7.1 ± 0.11 (32)	
B	*ND*	8.2 ± 0.14 (8)	7.3 ± 0.16 (4)	7.0 ± 0.092 (2)	6.6 ± 0.098 (2)	7.7 ± 0.18 (50)	< 4 (2)
C	6.0 ± 0.055 (2)	7.7 ± 0.10 (8)	6.7 ± 0.17 (4)	6.8 ± 0.096 (2)	6.1 ± 0.16 (2)	7.2 ± 0.16 (32)	
D	*ND*	7.7 ± 0.070 (4)	7.0 ± 0.044 (2)	6.8 ± 0.10 (2)	5.9 ± 0.087 (2)	7.2 ± 0.18 (34)	
**KDM3**[‡]							
A	< 4 (2)	6.0 ± 0.19 (4)	5.6 ± 0.14 (2)	*ND*	*ND*	4.5 ± 0.44 (20)	
B	*ND*	*ND*	*ND*	*ND*	*ND*	4.1 ± 0.65 (8)	
**KDM4**[‡]							
A	*ND*	6.2 ± 0.10 (2)	5.0 ± 0.13 (2)	*ND*	*ND*	5.0 ± 0.69 (6)	
C	5.9 ± 0.63 (2)	6.8 ± 0.14 (6)	5.6 ± 0.21 (4)	*ND*	*ND*	5.3 ± 0.28 (12)	
E	5.0 ± 0.16 (2)	*ND*	*ND*	*ND*	*ND*	*ND*	
**KDM6B**[‡]	< 4 (2)	5.9 ± 0.10 (2)	5.0 ± 0.13 (2)	5.1 ± 0.14 (2)	ND	4.0 ± 1.1 (6)	
**KDM2A**[‡]	4.3 ± 0.50 (2)	5.7 ± 0.050 (2)	4.7 ± 0.15 (2)	*ND*	*ND*	4.4 ± 0.86 (4)	
**PHF8**[‡]	*ND*	*ND*	*ND*	*ND*	*ND*	5.0 ± 0.057 (2)	
**MINA53**[#]						< 4 (2)	
**NO66**[#]						< 4 (2)	
**FIH**[§]						< 4 (2)	
**EGLN1**[§]						< 4 (2)	

* pIC50 values ± SEM (number of independent values); [‡] AlphaScreen assay; [#] RapidFire MS assay; [§] MALDI-TOF assay.

Table S2 related to Figure 2 Crystallographic Statistics for the Structure of the KDM5B/KDOAM-25 Complex

	KDM5B KDOAM-25 (5a3n)
**Data collection**	
Space group	$P6_522$
Cell dimensions	
a, b, c (Å)	141.87, 141.87, 151.86
α, β, γ (°)	90, 90, 120
Resolution (Å)	19.81 - 2.0
	(2.07 - 2.00)
R_{merge}	0.08047 (0.6908)
$I / \sigma I$	22.81 (2.99)
Completeness (%)	99.29 (93.57)
Redundancy	12.6 (8.4)
**Refinement**	
Resolution (Å)	2.00
No. reflections	763080 (47111)
R_{work} / R_{free}	0.1803/0.2353
No. atoms	
Protein	3671
Ligand/ion	83
Water	238
B-factors	
Protein	36.60
Ligand/ion	43.50
Water	43.30
R.m.s deviations	
Bond lengths (Å)	0.008
Bond angles (°)	1.15

Data were collected from one crystal for each structure.*Values in parentheses are for highest-resolution shell.

Table S3 related to Figure 1 Caco-2 cell permeability assay and Liver microsomes stability of KDOAM-20, -21 and -25

	KDOAM-25	KDOAM-21	KDOAM-20
**Caco-2 A-B**	<0.76	5.3	0.76
**Caco-2 B-A**	<0.76	15	0.25
**PAMPA pH 5**	<0.3	<0.2	<0.13
**PAMPA pH 6.5**	<0.23	0.233	<0.20
**PAMPA pH 7.4**	<0.23	<0.4	<0.20
**HLM Clint**	*11.9 μL/min/mg*	*ND*	*ND*
**MLM Clint**	*8.42 μL/min/mg*	*ND*	*ND*
**RLM Clint**	*<1 μL/min/mg*	*ND*	*ND*

Table S4 related to Figure 1 KDOAM-21 and -25 chemical stability in Phosphate Buffer Saline

	**KDOAM-21**		**KDOAM-25**	
	Relative % at 254 nm		Relative % at 254 nm	
Time	Ester	Acid	Amide	Acid
0 min	100	0	100	0
20 min	100	0	100	0
1 hour	100	0	100	0
4 hours	93	7	100	0
24 hours	75	25	100	0

Table S5 related to Figure 1 KDOAM-25 Activity in the Cerep Express Panel

Assay	% Inhibition of reference (10 µM)	Reference Compound	K_i Ref (nM)
A1 (h) (antagonist radioligand)	-3	DPCPX	0.56
A2A (h) (agonist radioligand)	-2	NECA	21
A3 (h) (agonist radioligand)	-6	IB-MECA	0.19
alpha 1 (non-selective) (antagonist radioligand)	-15	prazosin	0.1
alpha 2 (non-selective) (antagonist radioligand)	-11	yohimbine	37
beta 1 (h) (agonist radioligand)	5	atenolol	230
beta 2 (h) (agonist radioligand)	5	ICI 118551	0.17
AT1 (h) (antagonist radioligand)	-7	saralasin	0.46
BZD (central) (agonist radioligand)	-42	diazepam	5.9
B2 (h) (agonist radioligand)	-10	NPC 567	9
CB1 (h) (agonist radioligand)	-1	CP 55940	1.3
CCK1 (CCKA) (h) (agonist radioligand)	-3	CCK-8s	0.22
D1 (h) (antagonist radioligand)	10	SCH 23390	0.19
D2S (h) (antagonist radioligand)	19	(+)butaclamol	0.73
ETA (h) (agonist radioligand)	-6	endothelin-1	0.015
GABA (non-selective) (agonist radioligand)	-4	GABA	14
GAL2 (h) (agonist radioligand)	-4	galanin	0.55
CXCR2 (IL-8B) (h) (agonist radioligand)	5	IL-8	0.058
CCR1 (h) (agonist radioligand)	-8	MIP-1alpha	0.042
H1 (h) (antagonist radioligand)	1	pyrilamine	1.5
H2 (h) (antagonist radioligand)	-16	cimetidine	510
MC4 (h) (agonist radioligand)	5	NDP-alpha -MSH	0.42
MT1 (ML1A) (h) (agonist radioligand)	5	melatonin	0.48
M1 (h) (antagonist radioligand)	-18	pirenzepine	18
M2 (h) (antagonist radioligand)	-3	methoctramine	29
M3 (h) (antagonist radioligand)	5	4-DAMP	1
NK2 (h) (agonist radioligand)	3	[Nleu10]-NKA (4-10)	1.5
NK3 (h) (antagonist radioligand)	-8	SB 222200	9.9
Y1 (h) (agonist radioligand)	-7	NPY	0.1
Y2 (h) (agonist radioligand)	-1	NPY	0.029
NTS1 (NT1) (h) (agonist radioligand)	-12	neurotensin	0.35
delta 2 (DOP) (h) (agonist radioligand)	2	DPDPE	1.5
kappa (KOP) (agonist radioligand)	-3	U 50488	0.64
mu (MOP) (h) (agonist radioligand)	-2	DAMGO	0.25

Assay	% Inhibition of reference (10 µM)	Reference Compound	K_i Ref (nM)
NOP (ORL1) (h) (agonist radioligand)	-5	nociceptin	0.11
EP4 (h) (agonist radioligand)	-15	PGE2	0.16
5-HT1A (h) (agonist radioligand)	-7	8-OH-DPAT	0.21
5-HT1B (antagonist radioligand)	-1	serotonin	6.9
5-HT2A (h) (antagonist radioligand)	-6	ketanserin	0.44
5-HT2B (h) (agonist radioligand)	17	(±)DOI	2.7
5-HT3 (h) (antagonist radioligand)	8	MDL 72222	4.8
5-HT5a (h) (agonist radioligand)	-24	serotonin	66
5-HT6 (h) (agonist radioligand)	-3	serotonin	72
5-HT7 (h) (agonist radioligand)	-10	serotonin	0.16
sst (non-selective) (agonist radioligand)	0	somatostatin-14	0.2
VPAC1 (VIP1) (h) (agonist radioligand)	-12	VIP	0.27
V1a (h) (agonist radioligand)	-4	[d(CH2)51,Tyr(Me)2]-AVP	1.2
Ca2+ channel (L, verapamil site) (phenylalkylamine) (antagonist radioligand)	-1	D 600	18
KV channel (antagonist radioligand)	-4	alpha -dendrotoxin	0.22
SKCa channel (antagonist radioligand)	5	apamin	0.0048
Na+ channel (site 2) (antagonist radioligand)	7	veratridine	7400
Cl- channel (GABA-gated) (antagonist radioligand)	-2	picrotoxinin	130
norepinephrine transporter (h) (antagonist radioligand)	-6	protriptyline	2.6
dopamine transporter (h) (antagonist radioligand)	13	BTCP	5.6
5-HT transporter (h) (antagonist radioligand)	-2	imipramine	1.1

Figure S1 related to Figure 2 Inhibition of KDM4/5 mediated H3K4me3 demethylation in cells

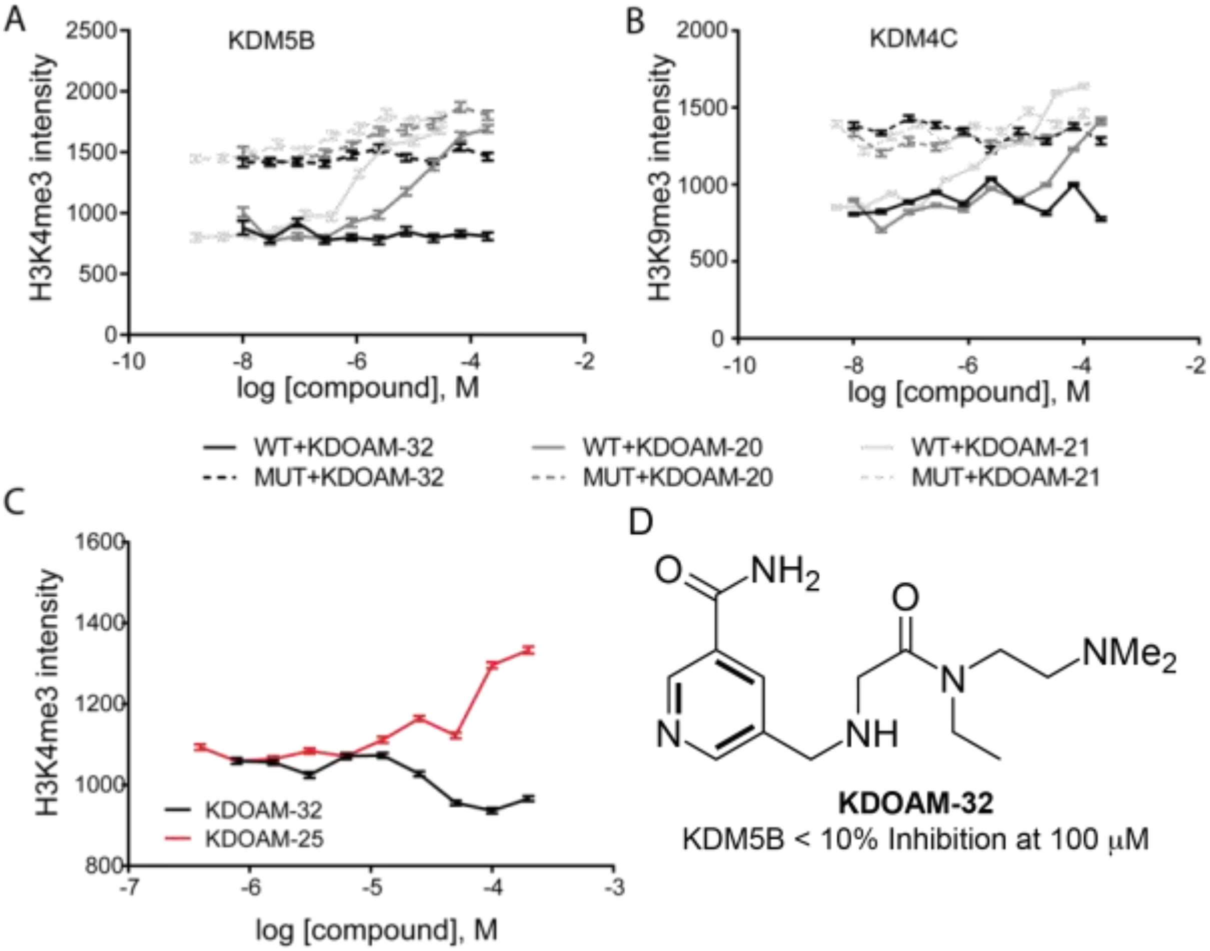

(A) EC_{50} curves of KDOAM-20, KDOAM-21 and KDOAM-32 in KDM5B and KDM4C (B) transfected HeLa cells (WT = wildtype; MUT = inactive mutant). (C) EC_{50} curves of KDOAM-25 and KDOAM-32 in untransfected HeLa cells. (D) KDOAM-32 is an inactive Control for KDOAM-25.

Figure S2 related to Figure 1 KDM5B Biochemical assay at different 2-OG concentrations

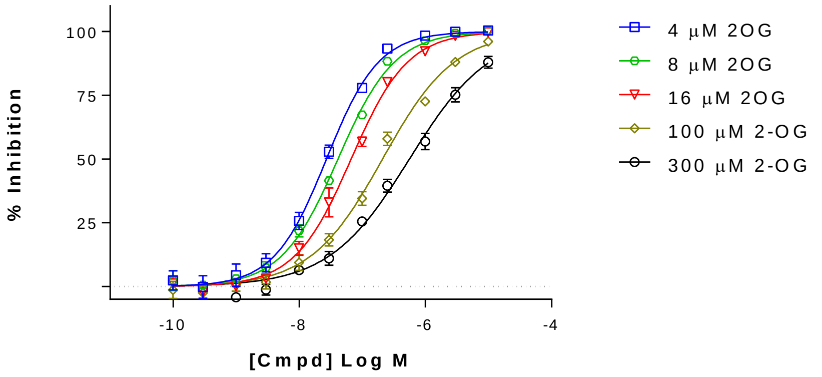

2OG Concentration	IC50 (µM)	K_i
4 µM 2OG	0.027	0.005
8 µM 2OG	0.041	0.005
16 µM 2OG	0.066	0.005
100 µM 2OG	0.210	0.003
300 µM 2OG	0.558	0.003

Figure S3 related to Figure 4 KDOAM-25 cytotoxicity

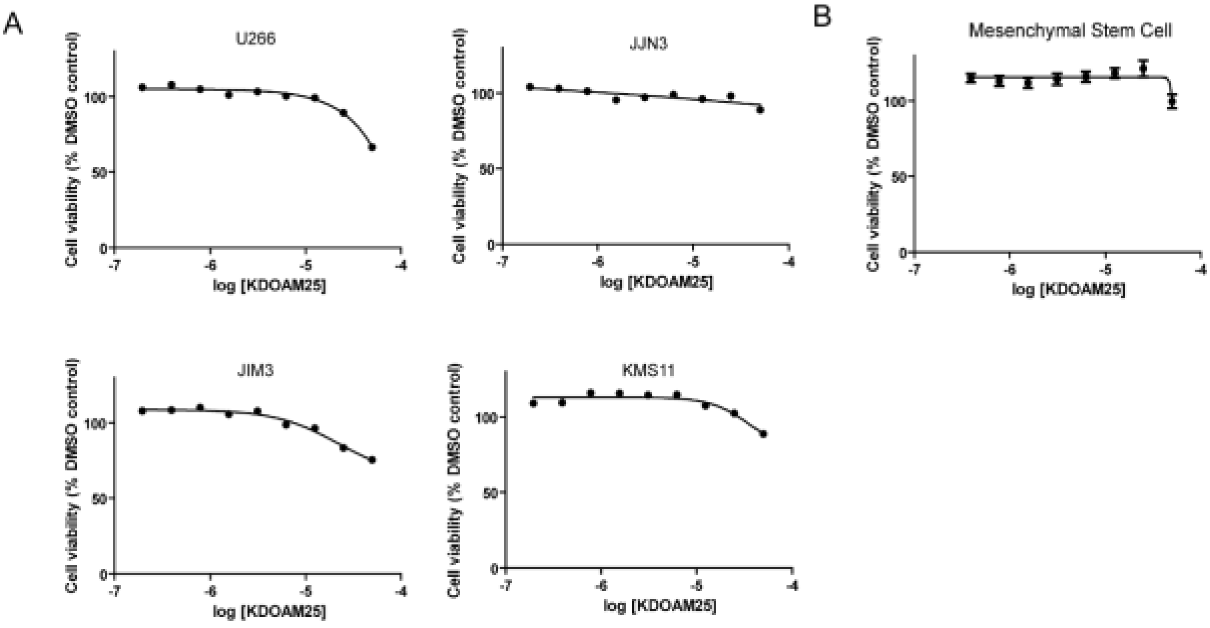

KDOAM-25 does not decrease cell viability in a range of multiple myeloma cell lines (A) or Mesenchymal Stem Cells (B) treated for 7 days. Data plotted represents the mean and standard deviation of four independent experiments.

Supplemental Experimental Procedures

General Experimental for Chemical Synthesis

Reactions were monitored using thin layer silica gel chromatography (TLC) 0.25 mm silica gel 60F plates from Merck. Plates were visualized by irradiation under UV lamp. Products were purified by flash chromatography on Biotage Isolera system using prepacked SNAP-Ultra, amino-functionalised or KP-Sil silica columns. Mass spectra were recorded using a Micromass Platform 1 spectrometer, operating in the positive or negative ion mode or HPLC-MS measurements were carried out using a Waters Autopurification System equipped with Waters 2489 UV/Vis detector, Waters 2424 ELS detector and SQ Detector 2. Chromolite Performance RP-18e, 3x100 mm and Phenomenex Kinetex 5µ EVO C18 100A, 3x100 mm columns were used for the analytical measurements using a gradient program; Chromolite SemiPrep RP18-e 10x100 mm and Phenomenex 5µ EVO C18 100A 21.2x150 mm columns were used for the preparative HPLC purifications using a gradient program; Eluent I: acetonitrile/water = 5/95 with 20 mM ammonium acetate buffer, pH 6.0, Eluent II: acetonitrile/water = 80/20 with 20 mM ammonium acetate buffer, pH 6.0. LCMS measurements were carried out using a WATERS sunfire C18 column using electrospray ionization, operating in the positive ion mode; separation was achieved using a linear gradient of solvent A (water + 0.01% CF_3CO_2H) and solvent B (acetonitrile + 0.01% CF_3CO_2H), eluting at a flow rate of 1 mL/min and monitoring at 254 nm: 0% B over 2 min, 0% B to 100% B over 16 min and 100% B over 2 min. NMR experiments were run on a Bruker Avance III 400 system (400.1 MHz for ^{1}H, 100.6 MHz for ^{13}C) equipped with a BBI probe and Z-gradients or a Bruker AV400 system (400.2 MHz for ^{1}H, 100.6 MHz for ^{13}C). Spectra were acquired at 300 K using deuterated dimethyl sulfoxide (d6-DMSO), deuterated chloroform ($CDCl_3$) or deuterated methanol (CD_3OD) as solvents and calibrated on the residual non-deuterated solvent signal. High resolution mass spectra (HRMS) were recorded using Bruker MicroTOF internally calibrated with polyalanine. Elemental analyses were recorded by the elemental analysis service of London Metropolitan University.

Synthesis of KDOAM-1

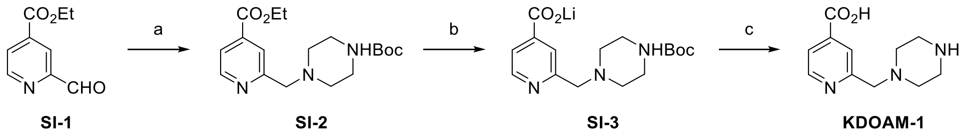

Reagents and conditions (a) *tert*-butyl piperazine-1-carboxylate, $NaBH(AcO)_3$, AcOH, CH_2Cl_2, quant.; (b) LiOH (aq), MeCN, quant.; (c) trifluoroacetic acid (TFA), 48%.

***tert*-Butyl 4-[(4-(Ethoxycarbonyl)pyridin-2-yl)methyl]piperazine-1-carboxylate (SI-2)**

tert-Butyl piperazine-1-carboxylate (1.15 g, 6.20 mmol) was added to a solution of ethyl 2-formylisonicotinate **SI-1** (Bavetsias et al., 2016) (735 mg, 4.10 mmol) in CH_2Cl_2 (20 mL) and the resulting solution stirred at room temperature for 1 h. Glacial acetic acid (3 drops) was added followed by $NaBH(AcO)_3$ (1.31 g, 6.20 mmol) and the resulting suspension stirred at room temperature for 16 h. The reaction mixture was diluted with water (20 mL), neutralised with saturated aq. sodium bicarbonate and extracted with CH_2Cl_2 (2 × 20 mL). The organic layers were washed with brine (20 mL) then combined, dried over magnesium sulfate and concentrated *in vacuo* to give **SI-2** as a colourless oil (1.44 g, quant.). R_f 0.30 (97.5:2.5 dichloromethane–methanol); LCMS (ES+): RT 9.6 min; *m/z* 350 $[M+H]^+$; ^{1}H-NMR (400 MHz, $CDCl_3$) 1.35–1.46 (12H, m), 2.42–2.48 (4H, m), 3.42–3.48 (4H,

m), 3.72 (2H, s), 4.39 (2H, q, *J* 7.0), 7.71 (1H, dd, *J* 5.0, 1.5), 7.92 (1H, app. s), 8.69 (1H, d, *J* 5.0); ^{13}C-NMR (125 MHz, $CDCl_3$) 14.1, 28.3, 42.8, 43.8, 52.9, 61.8, 64.1, 79.6, 121.4, 122.5, 138.2, 150.0, 154.7, 159.1, 165.2; HRMS: found 372.1891, calc. for $C_{18}H_{27}N_3NaO_4^+$ 372.1894.

Lithium 2-[(4-(*tert*-Butoxycarbonyl)piperazin-1-yl)methyl]isonicotinate (SI-3)

Lithium hydroxide (1 M, aq, 0.20 mL, 0.20 mmol) was added to a solution of *tert*-butyl 4-[(4-(ethoxycarbonyl)pyridin-2-yl)methyl]piperazine-1-carboxylate **SI-2** (70 mg, 0.20 mmol) in acetonitrile (1.0 mL) and water (0.8 mL) and the resulting solution stirred at room temperature for 16 h. The reaction mixture was concentrated *in vacuo* and the residue washed with cyclohexane (3 × 1 mL) to give **SI-3** as a yellow solid (68 mg, quant.). LCMS (ES+): RT 8.6 min (94%); *m/z* 322 $[M+H]^+$; ^{1}H-NMR (400 MHz, CD_3OD) 1.46 (9H, s), 2.45–2.52 (4H, m), 3.44–3.51 (4H, m), 3.71 (2H, s), 7.74 (1H, d, *J* 5.0), 7.97 (1H, s), 8.52 (1H, d, *J* 5.0); ^{13}C-NMR(100 MHz, CD_3OD) 28.8, 44.3, 45.3, 54.2, 64.9, 81.3, 123.5, 124.6, 148.5, 149.8, 156.5, 159.4, 172.6; HRMS: found 344.1577, calc. for $C_{16}H_{23}N_3NaO_4^+$ 344.1581

2-(Piperazin-1-ylmethyl)isonicotinic Acid Bis(trifluoroacetate) Monohydrate (KDOAM-1)

A solution of lithium 2-[(4-(*tert*-butoxycarbonyl)piperazin-1-yl)methyl]isonicotinate **SI-3** (61 mg, 0.19 mmol) in trifluoroacetic acid (1.0 mL) was stirred at room temperature for 16 h. The reaction mixture was concentrated *in vacuo* and the residue purified by strong-cation exchange chromatography, washing with methanol and eluting with methanolic ammonia (5% v/v solution of conc. ammonia in methanol) to give an off-white solid that was taken up in acetonitrile (1 mL). Trifluoroacetic acid (35 µL, 0.46 mmol) was added, the resulting suspension filtered and the filtrate concentrated *in vacuo*, azeotroping with water (2 × 2 mL) followed by ethyl acetate (2 × 2 mL) to give **KDOAM-1** as a beige gum (43 mg, 48%). LCMS (ES+): RT 1.9 min; *m/z* 222 $[M+H]^+$; ^{1}H-NMR (400 MHz, CD_3OD) 3.11–3.22 (4H, m), 3.39–3.48 (4H, m), 4.22 (2H, s), 7.98 (1H, d, *J* 5.0), 8.13 (1H, s), 8.79 (1H, d, *J* 5.0); ^{13}C-NMR (100 MHz, CD_3OD) 43.8, 50.7, 62.2, 124.7, 125.3, 142.7, 150.3, 156.4, 167.1; HRMS: found 222.1232, calc. for $C_{11}H_{16}N_3O_2^+$ 222.1237; elemental analysis: $C_{15}H_{19}F_6N_3O_7$ requires C, 38.55; H, 4.1; N, 9.0%; found C, 38.4; H, 3.9; N, 9.1%.

Synthesis of KDOAM-21

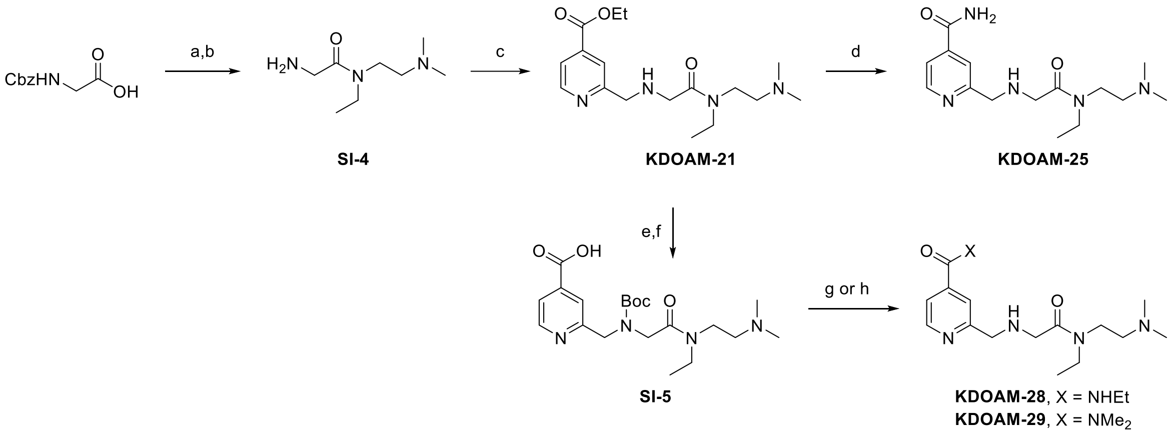

Reagents and conditions (a) *N*-ethyl-*N'*,*N'*-dimethylethane-1,2-diamine, T3P, TEA, CH_2Cl_2, 64%; (b) cyclohexene, Pd/C, EtOH, 90%.; (c) **SI-1**, $NaBH(AcO)_3$, DCE, 75%; (d) NH_3, MeOH, 82%; (e) Boc_2O, TEA, DCE, 52%; (f) KOH, THF/MeOH/H_2O, 64%; (g) i. $EtNH_2$, HATU, DBU, CH_2Cl_2, 53%, ii. HCl, dioxane, 64%; (h) i. Me_2NH, HATU, DBU, CH_2Cl_2, 48%, ii. HCl, dioxane, 76%.

2-amino-*N*-(2-(dimethylamino)ethyl)-*N*-ethylacetamide (SI-4)

Step 1, benzyl (2-((2-(dimethylamino)ethyl)(ethyl)amino)-2-oxoethyl)carbamate: ((Benzyloxy)carbonyl)-glycine (6 g, 28.7 mmol) was dissolved in anhydrous CH_2Cl_2 (25 mL), then 2,4,6-tripropyl-1,3,5,2,4,6-trioxatriphosphinane 2,4,6-trioxide (T3P) (27.9 ml, 43.0 mmol) and triethylamine (TEA) (6.00 ml, 43.0 mmol) were sequentially added. After 10 minutes, *N*-ethyl-*N',N'*-dimethylethane-1,2-diamine (4.52 ml, 28.7 mmol) was added. The solution was stirred at room temperature for 16 h, then diluted with CH_2Cl_2 and washed with 1N NaOH. The aqueous phase was extracted with additional CH_2Cl_2 (2 x 30 mL).The organic phase was dried over Na_2SO_4, filtered and concentrated to dryness. Product was purified on Biotage Isolera by silica chromatography with eluting system CH_2Cl_2/MeOH (from 100:0 to 80:20). (5.6 g, 64%) was recovered in pure form. LCMS (ES+): RT = 1.88min; m/z = 308 $[M-H]^+$; ^{1}H-NMR (d_6-DMSO): δ = 7.40-7.29 (m, 5H), 7.24 (t, 1H, J = 5.7 Hz), 5.04 (s, 2H), 3.86 (dd, 2H, J = 5.7, 15.0 Hz), 3.33-3.25 (m, 4H), 2.35 (m, 2H), 2.20-2.12 (m, 6H), 1.06 (m, 3H) ppm.

Step 2: Benzyl (2-((2-(dimethylamino)ethyl)(ethyl)amino)-2-oxoethyl)carbamate (6.6 g, 21.47 mmol) was dissolved in EtOH (350 mL), then cyclohexene (43.5 mL, 429 mmol) and palladium on activated charcoal (5.09 g, 4.29 mmol) were added in one portion. The solution was degassed (vacuum/N_2 cycles, 3 times) and stirred at 60 °C for 2 h. The solution was then filtered through a pad of Celite and the organic phase was concentrated to dryness. The product (5.7g, 90%) was pure enough for further elaborations. LCMS (ES+): RT = 0.34min; m/z = 174 $[M-H]^+$; ^{1}H-NMR (d_6-DMSO): δ = 5.06 (bs, 4H), 3.52-3.20 (m, 6H), 2.45-2.28 (m, 2H), 2.22-2.12 (m, 6H), 1.88 (s, 6H), 1.13-0.99 (m, 3H) ppm.

Ethyl 2-(((2-((2-(Dimethylamino)ethyl)(ethyl)amino)-2-oxoethyl)amino)methyl)isonicotinate (KDOAM-21)

A solution of ethyl 2-formylisonicotinate (**SI-1**) (0.69 g, 3.85 mmol) and 2-amino-*N*-(2-(dimethylamino)ethyl)-*N*-ethylacetamide (**SI-4**) (1.695 g, 5.78 mmol) in 1,2-dichloroethane (DCE) (80 mL) was stirred at room temperature for 15 min, then $NaBH(AcO)_3$ (1.306 g, 6.16 mmol) was added in one portion. The solution was stirred at room temperature for 16 h, then the solvent was evaporated. Purification on Biotage Isolera by RP silica chromatography with eluting system H_2O/MeCN from 95:5 to 0:100, afforded a first crop of product (0.900g, 51%) as double acetate salt, and a second crop of product (0.316g, 24%) as free base. LCMS (ES+): RT =1.04min; m/z = 337 $[M-H]^+$; Free base, mixture of rotamers: ^{1}H NMR (400 MHz, Methanol-d_4) δ 8.71 (dd, J = 5.1, 0.9 Hz, 1H), 8.01 (dd, J = 1.6, 0.8 Hz, 1H), 7.82 (dd, J = 5.1, 1.6 Hz, 1H), 4.44 (q, J = 7.1 Hz, 2H), 4.02 (s, 2H), 3.58 (s, 2H), 3.56 – 3.34 (m, 2H), 2.63 – 2.45 (m, 4H), 2.32 (d, J = 38.9 Hz, 6H), 1.42 (t, J = 7.1 Hz, 3H), 1.16 (dt, J = 21.6, 7.1 Hz, 3H); ^{13}C NMR (101 MHz, Methanol-d_4) δ 170.79, 164.87, 160.10, 149.57, 138.81, 121.47, 121.29, 61.61, 57.08, 56.07, 53.41, 48.60, 44.36, 44.12, 42.56, 41.58, 13.07, 12.65, 11.69.

Synthesis of KDOAM-25

2-(((2-((2-(Dimethylamino)ethyl)(ethyl)amino)-2-oxoethyl)amino)methyl)isonicotinamide (KDOAM-25)

The ethyl ester, **KDOAM-21** (0.3 g, 0.657 mmol), was dissolved in a solution of ammonia in methanol (7 M, 15.02 mL, 105 mmol) and the mixture was stirred at room temperature for 20h. After evaporation of the solvent, the crude was purified by Biotage Isolera automated chromatography (NH_2-Si cartridge, 11g) with eluting system CH_2Cl_2/MeOH from 100:0 to 95:5, affording pure product (0.165 g, 82%). The HCl salt was formed by dissolving the free base in a solution of HCl in dioxane (4M, 2 mL, 8 mmol) and the mixture was stirred at room

temperature for 6h. After evaporation of solvent and lyophilisation, the product (0.014 g, quant.) was recovered in pure form as an amorphous solid. LCMS (ES-/+): RT = 0.60 min; m/z = 306 $[M-H]^-$; m/z = 308 $[M-H]^+$; ^{1}H-NMR (CD_3OD): δ = 8.69 (dd, 1H, J = 0.5, 5.1 Hz), 7.77 (s, 1H), 7.71 (dd, 1H, J = 1.5, 5.1 Hz), 4.47 (s, 2H), 4.20 (s, 2H), 3.74 (t, 2H, J = 6.0 Hz), 3.35-3.26 (m, 4H), 2.88 (s, 6H), 1.16 (t, 3H, J = 7.1 Hz) ppm. ^{13}C-NMR (CD_3OD): δ = 167.7, 166.7, 151.9, 149.5, 143.2, 121.5, 120.9, 55.4, 50.0, 42.8, 41.5, 39.8, 12.1 ppm. HRMS: found 308.2078, calc. for $C_{15}H_{26}N_5O_2^+$ 308.2081.

Synthesis of KDOAM-28

2-(((*tert*-Butoxycarbonyl)(2-((2-(dimethylamino)ethyl)(ethyl)amino)-2-oxoethyl)amino)-methyl)iso-nicotinic acid (SI-5)

Step 1, Ethyl 2-(((*tert*-Butoxycarbonyl)(2-((2-(dimethylamino)ethyl)(ethyl)amino)-2-oxoethyl)amino)-methyl)isonicotinate: To a solution of **KDOAM-21** (0.45 g, 1.338 mmol) in CH_2Cl_2 (135 mL), di-tert-butyl dicarbonate (Boc_2O) (0.321 g, 1.471 mmol) and TEA (0.559 mL, 4.01 mmol) were sequentially added in one portion. The solution was stirred at room temperature for 1.5 h. After evaporation of the solvent, the crude product was purified by Biotage Isolera automated RP chromatography (C_{18} cartridge, 12 g) with eluting system H_2O/MeCN from 95:5 to 0:100, affording pure product (0.300 g, 51%). LCMS (ES+): RT = 2.25min; *m/z* = 437 $[M-H]^+$; ^{1}H-NMR (d_6-DMSO): δ = 8.73-8.70 (m, 1H), 7.81-7.70 (m, 2H), 4.57-4.47 (m, 2H), 4.41-4.32 (m, 2H), 4.22-4.04 (m, 2H), 3.30-3.19 (m, 4H), 2.38-2.30 (m, 2H), 2.21-2.05 (m, 6H), 1.41-1.23 (m, 12H), 1.14-0.97 (m, 3H) ppm.

Step 2: The product of the previous reaction (0.300 g, 0.687 mmol) in a 1:1:1 mixture of THF/MeOH/H_2O (24 mL) was treated with KOH (0.116 g, 2.06 mmol). The solution was stirred at room temperature for 16 h, then the solution was adjusted to pH 3 with 1N HCl. After evaporation of solvents, the crude product was purified by Biotage Isolera automated RP chromatography (C_{18} cartridge, 12 g) with eluting system H_2O/MeCN from 95:5 to 0:100, affording pure product (0.18 g, 64%) as the double hydrochloric salt. LCMS (ES+): RT = 0.62 min; *m/z* = 307 $[M-H-Boc]^-$; *m/z* = 309 $[M+H-Boc]^+$; ^{1}H-NMR (d_6-DMSO): δ = 8.56-8.31 (m, 1H), 7.73-7.41 (m, 2H), 4.50-4.38 (m, 2H), 4.22-4.03 (m, 2H), 3.63-3.55 (m, 2H), 3.39-3.25 (m, 2H), 2.97-2.86 (m, 2H), 2.62-2.54 (m, 6H), 1.41-1.19 (m, 9H), 1.18-0.99 (m, 3H) ppm.

2-(((2-((2-(Dimethylamino)ethyl)(ethyl)amino)-2-oxoethyl)amino)methyl)-*N*-ethylisonicotinamide (KDOAM-28)

To a solution of **SI-5***2HCl (20 mg, 0.042 mmol) and 1,8-diazabicycloundec-7-ene (DBU) (6.21 µL, 0.042 mmol) in CH_2Cl_2 (2mL), 1-[bis(dimethylamino)methylene]-1H-1,2,3-triazolo[4,5-b]pyridinium 3-oxide hexafluorophosphate (HATU) (47.4 mg, 0.125 mmol) was added in one portion. After 5 min of stirring, ethylamine (2 M solution in THF, 31 µL, 0.062 mmol) was added in one portion. The solution was stirred at room temperature for 20 h. After evaporation of the solvent, the crude product was purified by Biotage Isolera automated RP chromatography (C_{18} cartridge, 12g) with eluting system H_2O/MeCN from 95:5 to 0:100, affording pure product (0.013 g, 72%). LCMS (ES+): Rt = 1.92 min; *m/z* = 436 $[M-H]^+$.

To a solution of the purified product from the previous reaction (0.013 g, 0.030 mmol), hydrogen chloride in 1,4-dioxane (1.5 ml, 0.030 mmol) was added in one portion, followed by addition of water (0.5 mL) and a drop of 37% HCl solution. The reaction was stirred at room temperature for 3 h. After evaporation of the solvent, the crude product was purified by Biotage Isolera automated RP chromatography (C_{18} cartridge, 12g) with eluting

system H_2O/MeCN from 95:5 to 0:100, affording pure product (0.007 g, 53%) in pure form. LCMS (ES+): RT = 1.63min; *m/z* = 336 $[M-H]^+$; ^{1}H-NMR (CD_3OD): δ = 8.60-8.67 (m, 1H), 7.76 (bs, 1H), 7.69-7.66 (m, 1H), 4.48 (s, 2H), 4.21 (s, 2H), 3.77-3.72 (m, 2H), 3.38-3.28 (m, 6H), 2.89 (bs, 6H), 1.25-1.11 (m, 6H) ppm. ^{13}C-NMR (101 MHz, d_6-DMSO) δ 166.57, 164.36, 153.12, 149.96, 143.07, 134.68, 121.47, 53.92, 50.69, 47.59, 45.72, 42.86, 41.30, 34.67, 15.04, 13.70, 8.87.

Synthesis of KDOAM-29

To a solution of **SI-5** (30 mg, 0.062 mmol) and DBU (0.023 ml, 0.156 mmol) in CH_2Cl_2 (2mL), HATU (71 mg, 0.187 mmol) was added in one portion. After 5 minutes of stirring, dimethylamine*HCl (7.62 mg, 0.093 mmol) was added in one portion. The solution was stirred at room temperature for 2 0h. After evaporation of the solvent, the crude was purified by Biotage Isolera automated RP chromatography (C_{18} cartridge, 12 g) with eluting system H_2O/MeCN from 95:5 to 0:100, affording pure product (0.013g, 48%) in pure form. RT = 1.92 min; ESI-MS: *m/z* = 436 $[M-H]^+$. To a solution of the purified product from the previous reaction (30 mg, 0.059 mmol), hydrogen chloride in 1,4-dioxane (1.5 ml, 0.030 mmol) was added in one portion, followed by addition of water (0.5 mL) and a drop of 37% HCl solution. The reaction was stirred at room temperature for 3 h. After evaporation of the solvent, the crude was purified by Biotage Isolera automated RP chromatography (C_{18} cartridge, 12 g) with eluting system H_2O/MeCN from 95:5 to 0:100, affording pure product (0.02g, 76%) in pure form. LCMS (ES^+): RT = 0.28 min; *m/z* = 336 $[M-H]^+$; ^{1}H-NMR (CD_3OD): δ = 8.76 (dd, 1H, *J* = 0.5, 5.0 Hz), 7.52 (m, 1H), 7.47 (dd, 1H, *J* = 1.3, 5.0 Hz), 4.56 (s, 2H), 4.32 (s, 2H), 3.85 (t, 2H, *J* = 6.0 Hz), 3.46-3.38 (m, 4H), 3.15 (s, 3H), 3.00 (s, 9H), 1.28 (t, 3H, *J* = 6.0 Hz) ppm; ^{13}C-NMR (CD_3OD): δ = 167.7, 166.7, 151.9, 149.7, 143.2, 121.1, 120.1, 55.3, 50.1, 42.8, 41.5, 39.9, 38.2, 34.0, 12.1.

Synthesis of KDOAM-32

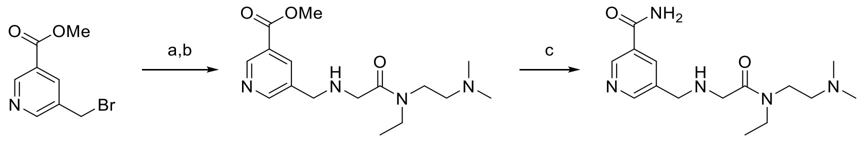

Reagents and conditions (a) $AgBF_4$, TEA, DMSO, 41%; (b) **SI-4**, $NaBH(AcO)_3$, DCE, 41%; (c) NH_3, MeOH, 66%.

Methyl 5-(((2-((2-(Dimethylamino)ethyl)(ethyl)amino)-2-oxoethyl)amino)methyl)nicotinate (SI-6)

Step 1: To a solution of methyl 5-(bromomethyl)nicotinate (200 mg, 0.869 mmol) in DMSO (5 mL) was added a solution of silver(I) tetrafluroborate (220 mg, 1.130 mmol) in dry DMSO (1 mL) . The suspension was stirred 40 min at RT, then TEA (0.364 mL, 2.61 mmol) was added and the mixture was stirred for further 15 min. The reaction was diluted with EtOAc and the organic solution was washed with sat. $NaHCO_3$ and brine. The aq. layer was further extracted with CH_2Cl_2 (2 x 20 mL) and EtOAc (2 x 20 mL). The combined organic fractions were dried (Na_2SO_4) and concentrated. The crude residue residue was purified by flash chromatography eluting with a gradient of 0 to 60% EtOAc in Cyclohexane. Methyl 5-formylnicotinate was obtained as white fluffy solid (60 mg, 41%). LCMS (ES+): RT 1.04 min; *m/z* 165 $[M+H]^{+\cdot}$ ^{1}H NMR (400 MHz, $CDCl_3$) δ 10.21 (s, 1H), 9.45 (d, *J* = 2.1 Hz, 1H), 9.27 (d, *J* = 2.1 Hz, 1H), 8.77 (t, *J* = 2.1 Hz, 1H), 4.03 (s, 3H).

Step 2: $NaBH(AcO)_3$ (90 mg, 0.424 mmol) was added to a solution of 2-amino-*N*-(2-(dimethylamino)ethyl)-*N*-ethylacetamide (**SI-4**) (118 mg, 0.678 mmol) and methyl 5-

formylnicotinate (70 mg, 0.424 mmol) in DCE (5 mL). The reaction was stirred for 3 h at RT. The solvent was removed under reduced pressure and the crude residue was purified by flash chromatography eluting with a gradient of 0 to 10% (MeOH + 1% NH_3). The title compound was isolated as colourless oil (56 mg, 41%). LCMS (ES+): RT 0.73, *m/z* 322 [M+H]+. ^{1}H-NMR (400 MHz, $CDCl_3$) δ 9.09 (d, J = 2.1 Hz, 2H), 8.73 (d, J = 2.2 Hz, 2H), 8.30 (t, J = 2.2 Hz, 2H), 3.98 – 3.84 (m, 7H), 3.51 (t, J = 7.1 Hz, 2H), 3.47 – 3.30 (m, 4H), 3.35 – 3.19 (m, 2H), 2.53 (t, J = 7.1 Hz, 2H), 2.38 – 2.16 (m, 6H), 1.13 (td, J = 7.1, 5.3 Hz, 4H).

5-(((2-((2-(Dimethylamino)ethyl)(ethyl)amino)-2-oxoethyl)amino)methyl)nicotinamide (KDOAM-32)

Ammonia (4.96 mL, 34.7 mmol) in Methanol was added to a sealed flask containing methyl 5-(((2-((2-(dimethylamino)ethyl)(ethyl)amino)-2-oxoethyl)amino)methyl)nicotinate (**SI-6**) (56 mg, 0.174 mmol). The slightly yellow solution was stirred at RT for 12 h until complete conversion was observed by LCMS. The solvents were removed under reduced pressure and the crude residue was dissolved in CH_2Cl_2 and purified by flash chromatography on a 11 g KP-NH column, eluting with a gradient of 0 to 10% MeOH in DCM. The desired product was isolated as colourless oil (35 mg, 66%). LCMS (ES+): RT 0.36, *m/z* 308 $[M+H]^+$, ^{1}H-NMR (400 MHz, $CDCl_3$) δ 8.91 (d, *J* = 2.2 Hz, 1H), 8.61 (d, *J* = 2.1 Hz, 1H), 8.15 (d, *J* = 2.2 Hz, 1H), 6.86 (s, 1H), 6.35 (s, 1H), 3.81 (s, 2H), 3.44 – 3.28 (m, 4H), 3.24 – 3.13 (m, 2H), 2.38 (d, *J* = 7.1 Hz, 1H), 2.32 (s, 3H), 2.19 (s, 3H), 2.14 (s, 3H), 1.06 (q, *J* = 7.2 Hz, 3H). ^{13}C-NMR (101 MHz, $CDCl_3$) δ 170.30, 167.75, 152.36, 147.76, 135.31, 129.01, 58.04, 56.95, 51.49, 49.37, 45.59, 43.47, 41.82, 41.38, 13.97, 12.94.

Stability of KDOAM-21 and KDOAM-25 in DPBS

HPLC-MS measurements were carried out using a Waters Autopurification System equipped with Waters 2998 photodiode array detector, Waters 2424 ELS detector and ACQUITY QDa Detector. A Phenomenex 5µ Gemini-NX C18 110A, 4.6 x 250 mm column was used for the analytical measurements using a gradient program; Eluent A: acetonitrile/water = 5/95 with 20 mM ammonium acetate buffer, pH 6.0, Eluent B: acetonitrile/water = 80/20 with 20 mM ammonium acetate buffer, pH 6.0, eluting at a flow rate of 2 mL/min and monitoring at 254 nm: 0% B over 2 min, 0% B to 95% B over 2.5 min and 95% B over 2 min.

1 mM solutions of KDOAM-21 and KDOAM-25 in Dulbecco's Phosphate Buffered Saline (Sigma D8537) were prepared from 50 mM DMSO stocks of KDOAM-21 and KDOAM-25. The resulting solutions were maintained at 37 C and aliquots removed for analysis by HPLC-MS at 0 min, 20 min, 1 hour, 4 hours and 24 hours. The stability of KDOAM-21 and KDOAM-25 to decomposition to KDOAM-20 was determined by comparison of the integrated area of the peaks at 254 nm.

References

Bankevich, A., Nurk, S., Antipov, D., Gurevich, A.A., Dvorkin, M., Kulikov, A.S., Lesin, V.M., Nikolenko, S.I., Pham, S., Prjibelski, A.D., *et al.* (2012). SPAdes: a new genome assembly algorithm and its applications to single-cell sequencing. J Comput Biol *19*, 455-477.

Bavetsias, V., Lanigan, R.M., Ruda, G.F., Atrash, B., McLaughlin, M.G., Tumber, A., Mok, N.Y., Le Bihan, Y.-V., Dempster, S., Boxall, K.J., *et al.* (2016). 8-Substituted Pyrido[3,4-d]pyrimidin-4(3H)-one Derivatives As Potent, Cell Permeable, KDM4 (JMJD2) and KDM5 (JARID1) Histone Lysine Demethylase Inhibitors. J Med Chem *59*, 1388-1409.

Hopkinson, R.J., Tumber, A., Yapp, C., Chowdhury, R., Aik, W., Che, K.H., Li, X.S., Kristensen, J.B.L., King, O.N.F., Chan, M.C., *et al.* (2013). 5-Carboxy-8-hydroxyquinoline is a broad spectrum 2-oxoglutarate oxygenase inhibitor which causes iron translocation. Chem Sci *4*, 3110-3117.

Johansson, C., Velupillai, S., Tumber, A., Szykowska, A., Hookway, E., Nowak, R.P., Strain-Damerell, C., Gileadi, C., Philpott, M., Burgess-Brown, N., *et al.* (2016). Novel inhibitor chemotypes for human JARID1B/KDM5B enzymes highlight essential structure-activity relationships with KDM4 and KDM6-type histone demethylases. Nat Chem Biol.

Lambert, J.P., Tucholska, M., Pawson, T., and Gingras, A.C. (2014). Incorporating DNA shearing in standard affinity purification allows simultaneous identification of both soluble and chromatin-bound interaction partners. J Proteomics *100*, 55-59.

Schulz, M.H., Weese, D., Holtgrewe, M., Dimitrova, V., Niu, S., Reinert, K., and Richard, H. (2014). Fiona: a parallel and automatic strategy for read error correction. Bioinformatics (Oxford, England) *30*, i356-363.
