# Supplementary material for: Potent and Selective KDM5 Inhibitor Stops Cellular Demethylation of H3K4me3 at Transcription Start Sites and Proliferation of MM1S Myeloma Cells
Source: Cell Chem Biol. 2017 Mar 16;24(3):371–80. doi: 10.1016/j.chembiol.2017.02.006 (PMC5361737; doi:10.1016/j.chembiol.2017.02.006)
Supplement: Document S2. Article plus Supplemental Information [file mmc2.pdf]

Article

Cell Chemical Biology

Potent and Selective KDM5 Inhibitor Stops Cellular Demethylation of H3K4me3 at Transcription Start Sites and Proliferation of MM1S Myeloma Cells

Graphical Abstract

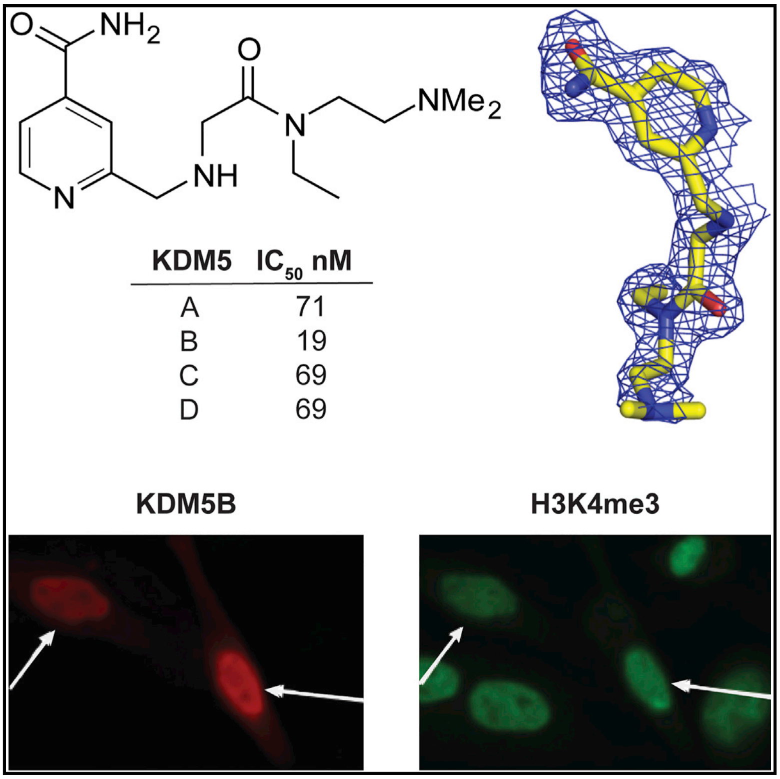

Authors

Anthony Tumber, Andrea Nuzzi,
Edward S. Hookway, ...,
Susanne Müller, Udo Oppermann,
Paul E. Brennan

Correspondence

susanne.muller-knapp@sgc.ox.ac.uk
(S.M.),
udo.oppermann@sgc.ox.ac.uk (U.O.),
paul.brennan@sgc.ox.ac.uk (P.E.B.)

In Brief

Tumber et al. report the discovery and characterization of KDOAM-25, a potent and selective inhibitor of the KDM5 H3K4me3 lysine demethylases. KDOAM-25 inhibits the cellular demethylation of H3K4me3 at transcription start sites and the proliferation of MM1S multiple myeloma cells.

Highlights

- KDOAM-25 is a selective inhibitor of KDM5A-D, the H3K4me3 lysine demethylases
- KDOAM-25 inhibits the demethylation of H3K4me3 in an immunofluorescence assay
- Increased KDM5B expression correlates with shorter survival in multiple myeloma
- KDOAM-25-treated cells show an increase in H3K4me3 at transcription start sites

Tumber et al., 2017, Cell Chemical Biology *24*, 371–380
March 16, 2017 © 2017 The Authors. Published by Elsevier Ltd.
http://dx.doi.org/10.1016/j.chembiol.2017.02.006

Please cite this article as: Tumber et al., Potent and Selective KDM5 Inhibitor Stops Cellular Demethylation of H3K4me3 at Transcription Start Sites and Proliferation of MM1S Myeloma Cells, Cell Chemical Biology (2017), http://dx.doi.org/10.1016/j.chembiol.2017.02.006

Potent and Selective KDM5 Inhibitor Stops Cellular Demethylation of H3K4me3 at Transcription Start Sites and Proliferation of MM1S Myeloma Cells

Anthony Tumber,[1,2,9] Andrea Nuzzi,[1,2,9] Edward S. Hookway,[3,9] Stephanie B. Hatch,[1,2,9] Srikannathasan Velupillai,[1,2,9] Catrine Johansson,[3,4] Akane Kawamura,[4,5] Pavel Savitsky,[1] Clarence Yapp,[1,2] Aleksandra Szykowska,[1] Na Wu,[3] Chas Bountra,[1] Claire Strain-Damerell,[1] Nicola A. Burgess-Brown,[1] Gian Filippo Ruda,[1,2] Oleg Fedorov,[1,2] Shonagh Munro,[6] Katherine S. England,[1,2] Radoslaw P. Nowak,[1,3] Christopher J. Schofield,[4] Nicholas B. La Thangue,[6] Charlotte Pawlyn,[7] Faith Davies,[7,8] Gareth Morgan,[7,8] Nick Athanasou,[3] Susanne Müller,[1,2,*] Udo Oppermann,[1,3,*] and Paul E. Brennan[1,2,10,*]

[1]Structural Genomics Consortium, University of Oxford, Oxford OX3 7DQ, UK
[2]Nuffield Department of Medicine, Target Discovery Institute, University of Oxford, Oxford OX3 7FZ, UK
[3]NIHR Oxford Biomedical Research Unit, Nuffield Department of Orthopedics, Rheumatology and Musculoskeletal Sciences, Botnar Research Centre, University of Oxford, Oxford OX3 7LD, UK
[4]Chemistry Research Laboratory, University of Oxford, 12 Mansfield Road, Oxford OX1 3TA, UK
[5]Division of Cardiovascular Medicine, Radcliffe Department of Medicine, University of Oxford, Oxford OX3 7BN, UK
[6]Department of Oncology, University of Oxford, Oxford OX3 7DQ, UK
[7]Division of Cancer Therapeutics, Institute of Cancer Research, Sutton, Surrey SM2 5NG, UK
[8]University of Arkansas for Medical Sciences, Myeloma Institute, 4301 W. Markham #816, Little Rock, AR 72205, USA
[9]Co-first author
[10]Lead Contact
*Correspondence: susanne.muller-knapp@sgc.ox.ac.uk (S.M.), udo.oppermann@sgc.ox.ac.uk (U.O.), paul.brennan@sgc.ox.ac.uk (P.E.B.)
http://dx.doi.org/10.1016/j.chembiol.2017.02.006

SUMMARY

Methylation of lysine residues on histone tail is a dynamic epigenetic modification that plays a key role in chromatin structure and gene regulation. Members of the KDM5 (also known as JARID1) sub-family are 2-oxoglutarate (2-OG) and Fe^{2+}-dependent oxygenases acting as histone 3 lysine 4 trimethyl (H3K4me3) demethylases, regulating proliferation, stem cell self-renewal, and differentiation. Here we present the characterization of KDOAM-25, an inhibitor of KDM5 enzymes. KDOAM-25 shows biochemical half maximal inhibitory concentration values of <100 nM for KDM5A-D in vitro, high selectivity toward other 2-OG oxygenases sub-families, and no off-target activity on a panel of 55 receptors and enzymes. In human cell assay systems, KDOAM-25 has a half maximal effective concentration of ~50 μM and good selectivity toward other demethylases. KDM5B is overexpressed in multiple myeloma and negatively correlated with the overall survival. Multiple myeloma MM1S cells treated with KDOAM-25 show increased global H3K4 methylation at transcriptional start sites and impaired proliferation.

INTRODUCTION

The site- and methylation state-specific modification of histone lysyl residues differentially regulates transcription by providing unique interaction sites that bind to methyl-lysine recognition (or ‘‘reader’’) domains (Ng et al., 2009). Genome-wide analyses of tri- or di-methylated histone 3- lysine 4 (H3K4me3 or H3K4me2) sites demonstrate that these histone marks are associated with promoter regions and transcriptional start sites at the 5′ end of genes (ENCODE Project Consortium, 2012). Interaction of these di- or tri-methylated histone marks with the transcription initiation factor TFIID (Lauberth et al., 2013), or with chromatin remodeling complexes, leads to formation of a pre-initiation complex that can be rapidly activated by further recruitment of specific transcription factors (Chai et al., 2013,; Tanny, 2014).

The dynamic interplay between chromatin methylation and demethylation is catalyzed by distinct classes of enzymes. The removal of methyl groups from histone lysine residues is catalyzed by two classes of lysine (K) demethylases (KDMs) with different co-factor and substrate dependencies: the flavine adenine dinucleotide-dependent monoamino oxidases (grouped as the KDM1 sub-family) and the Fe(II) and 2-oxoglutarate (2-OG)-dependent oxygenases that contain a conserved catalytic Jumonji C domain ([JmjC] belonging to sub-families KDM2- KDM7) (Johansson et al., 2014).

Members of the KDM5 sub-family of JmjC-KDMs are transcriptional co-repressors, which specifically catalyze the removal of all possible methylation states from lysine 4 of histone H3 (H3K4me3/me2/me1), with highest activity found toward H3K4me3. KDM5 enzymes are often found as components of transcriptional complexes with repressors such as REST, histone deacetylases, and histone methyl transferases (Pasini et al., 2008). In mammals the KDM5 sub-family encompasses four proteins, KDM5A (known as JARID1A or RBP2), KDM5B (known as JARID1B or PLU1), KDM5C (JARID1C or SMCX),

© 2017 The Authors. Published by Elsevier Ltd. This is an open access article under the CC BY license (http://creativecommons.org/licenses/by/4.0/).

and KDM5D (JARID1D or SMCY), the latter two encoded on the X and Y chromosomes, respectively.

The KDM5 family and domain architecture is conserved from yeast to humans, displaying a similar pattern with a Jumonji N domain (JmjN), a DNA binding ARID domain (AT-rich interactive domain), a catalytic JmjC, a C5HC2 zinc-finger motif located C-terminally to the JmjC, a PLU-1 motif, as well as one to three methyl-lysine or methyl-arginine binding plant homeodomains (PHDs) denoted PHD-1, PHD-2, and PHD-3. It is conceivable that these additional domains contribute critically to genomic KDM5 target gene occupation, since PHD domains are able to bind to modified lysine residues in a sequence-specific manner (Horton et al., 2016; Johansson et al., 2016).

The KDM5 enzymes are suggested to play pivotal roles both during normal development and in pathological conditions such as cancer. KDM5A is ubiquitously expressed and involved in the control of cell proliferation and differentiation, and linked to several human cancers including acute myeloid leukemia (Wang et al., 2009), hepatocellular carcinoma (Liang et al., 2013), gastric cancer (Li et al., 2014), (Zeng et al., 2010), and lung cancer (Teng et al., 2013; Wang et al., 2013). KDM5B plays an important role in stem cell biology by blocking differentiation in embryonic and hematopoietic stem cells (Cellot et al., 2013; Dey et al., 2008). Similar to KDM5A, elevated expression levels have been found in various primary cancers including melanoma (Roesch et al., 2010), breast (Barrett et al., 2002), testicular (Madsen et al., 2003), and ovarian cancer (Wang et al., 2015). KDM5C plays a role in neuronal development and shows highest expression in neuronal tissues. Inactivating mutations have been associated with X-linked intellectual disability (Santos-Reboucas et al., 2011). The Y chromosome-encoded KDM5D is expressed in all male tissues, and the protein has a suggested role in spermatogenesis (Akimoto et al., 2008).

Chemical tool compounds complement biological target identification strategies such as gene editing or knock down, and play important roles in dissecting the function of specific domains in target biology. Although inhibitors have been reported for JmjC-containing KDMs (Bavetsias et al., 2016; Gehling et al., 2016; Horton et al., 2016; Itoh et al., 2015; McAllister et al., 2016; Vinogradova et al., 2016; Westaway et al., 2016a, 2016b; Wu et al., 2016), most of the published compounds have limitations such as lack of selectivity or cytotoxicity. Bavetsias et al. (2016) reported a new class of cell-active histone lysine demethylase inhibitors. The compounds are based on a cell-permeable pyridopyrimidinone scaffold. However, these compounds do not show any selectivity between the KDM4 and KDM5 families. Another cell-active pan-KDM inhibitor is the pro-drug of IOX1 (Schiller et al., 2014), yet this compound is affected by high cytotoxicity. All these limitations hamper the use of these molecules as tool compounds in a cellular context. Among the others, a recently reported KDM5A inhibitor (Liang et al., 2016) is the most remarkable tool compound/pre-clinical candidate for cancer therapy. This orally bioavailable pyrazolopyrimidinone shows both high inhibitions of KDM5 demethylases on a TR-FRET biochemical assay and good activity in cell-based assays, thus making this compound the best chemical tool reported in the literature to date.

In this paper, we report the biochemical and molecular genome-wide characterization of KDOAM-25, a new, selective, and cell-active pan-KDM5 inhibitor that can be used to study H3K4 methylation biology.

RESULTS AND DISCUSSION

Discovery of KDOAM-25

We previously described 4-carboxy-2-triazolopyridines as selective KDM2 inhibitors (England et al., 2014). In efforts to find alternative scaffolds based on the 4-carboxypyridine core, a range of compounds with a 2-aminomethyl group were synthesized and tested for KDM inhibition. Aminomethylpyridines such as KDOAM-1 showed a preference for the KDM4 and KDM5 sub-families (Figure 1A), but we were unable to find analogs with a potency in cellular immunofluorescence (IF) assays of H3K36 and H3K4 demethylation. Hence, we were interested when a patent application described very similar compounds and reported potency in an almost identical cellular IF assay (Labelle et al., 2014). In our hands, two of the compounds described, named here KDOAM-20 and -21, showed low- and sub-micromolar potency, respectively, in an H3K4me3 IF assay overexpressing KDM5B, but also inhibited H3K9me3 demethylation in accordance with its in vitro KDM4 activity (Figures S1A and S1B). We considered it possible that KDOAM-21 may be acting as an ester pro-drug akin to the KDM5/6 inhibitor, GSK-J4 (Kruidenier et al., 2012). To our surprise, the ester KDOAM-21 itself showed potent inhibition of KDM5B in vitro (Figure 1A). However, KDOAM-20 was also a sub-micromolar inhibitor of KDM4C, and KDOAM-21 could potentially be hydrolyzed to KDOAM-20 in situ. Therefore, we sought to replace the ester of KDOAM-20 with a less labile group such as an amide to retain KDM5 potency and selectivity. There is some precedent for this, as compound **16** in Bavetsias et al. (2016) is a weak KDM4/5 inhibitor with some selectivity for KDM5A/B over KDM4A/B (5- to 50-fold). The amides KDOAM-25, -28, and -29 were synthesized, and KDOAM-25 was shown to be the most potent of the series with a KDM5B half maximal inhibitory concentration (IC_{50}) of 19 nM and improved selectivity over KDM4C.

We also investigated the biochemical inhibitory effect of KDOAM-25 at different concentrations of the co-factor 2-OG on KDM5B (Figure S2). The experiment indicated that the compound acts a partial competitor of the co-substrate 2-OG, with IC_{50} values increasing from 27 nM at 4 μM 2-OG, up to 558 nM at 300 μM 2-OG.

With a potent KDM5B inhibitor available in the form of KDOAM-25, we profiled it extensively for in vitro potency in all our available JmjC KDM assays and assays for representative 2-OG-dependent oxygenases (Figure 1B and Table S1) using AlphaScreen or MALDI-TOF enzymatic assays. KDOAM-25 was selective for the KDM5 sub-family with no inhibition below 4.8 μM for any of the other 2-OG oxygenases. KDOAM-25 was also profiled in the CEREP express panel of 55 common off-targets but showed no inhibition at 10 μM (Table S5).

KDOAM-25 Binds to KDM5B in the 2-OG and Substrate Sites

KDOAM-25 was crystallized in a JmjN-JmjC-linked construct of KDM5B (Johansson et al., 2016) (Figure 2). The pose of the compound was identical to the reported structure of KDOAM-20 in

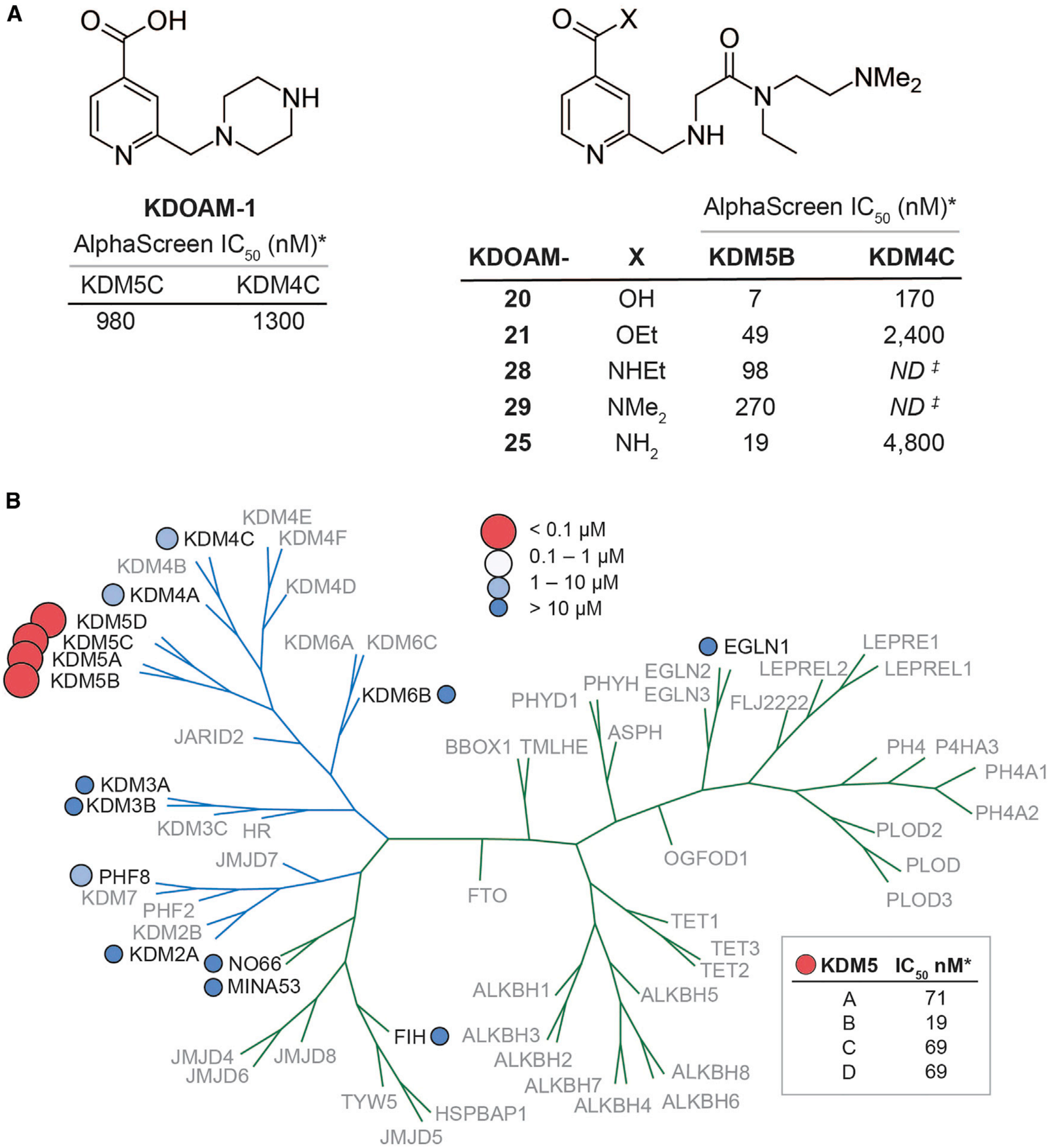

KDOAM-1

AlphaScreen IC_{50} (nM)*	
KDM5C	KDM4C
980	1300

KDOAM-	X	AlphaScreen IC_{50} (nM)* KDM5B	KDM4C
20	OH	7	170
21	OEt	49	2,400
28	NHEt	98	*ND* ‡
29	NMe_2	270	*ND* ‡
25	NH_2	19	4,800

KDM5	IC_{50} nM*
A	71
B	19
C	69
D	69

Figure 1. Discovery of KDOAM-25

(A) KDOAM-21 is a weak KDM4/5 inhibitor. KDOAM-20 and -25 are potent and selective inhibitors of KDM5B over KDM4C.

(B) KDOAM-25 was tested against a panel of 15 2-OG oxygenases (black text) including a representative from each of the lysine demethylases sub-families (blue branches). KDOAM-25 only inhibits the KDM5 sub-family members of KDM5A-D with potencies <100 nM (box).

*All IC_{50} values are an average of at least two independent determinations (Table S1); ‡Not Determined.

the same construct (PDB: 5A3T) with one key difference: in the carboxylate binding pocket, Y425 is shifted from an H-donor pose that interacts with the carboxylate of KDOAM-20 to allow an inverse H-bond interaction with the carboxamide of KDOAM-25. The shift of Y425 creates a cavity which is occupied by a water molecule. The propensity for Y425 to adopt this alternative pose in KDM5B may explain why KDOAM-25 is selective for KDM5 enzymes versus other KDMs that either form different H-bonds to the 4-carboxypyridines (KDM2/3/6/7) or have a less mobile homologous tyrosine (KDM4).

KDOAM-25 Increases H3K4me3 Levels in HeLa Cells

The cellular activity of KDOAM-25 was assessed in an IF assay by using an overexpression system, assessing the effect of KDOAM-25 and related compounds on the ectopically overexpressed wild-type (WT) demethylases compared with their catalytically dead mutants (MUT). As control we used a structurally related inactive compound, KDOAM-32 (Figure S1D), which is unable to coordinate the catalytic iron in the KDM5 active sites (Figure S1 and Table S1). Consistent with its in vitro activity, KDOAM-25 inhibited most potently KDM5B with an IC_{50}

Please cite this article as: Tumber et al., Potent and Selective KDM5 Inhibitor Stops Cellular Demethylation of H3K4me3 at Transcription Start Sites and Proliferation of MM1S Myeloma Cells, Cell Chemical Biology (2017), http://dx.doi.org/10.1016/j.chembiol.2017.02.006

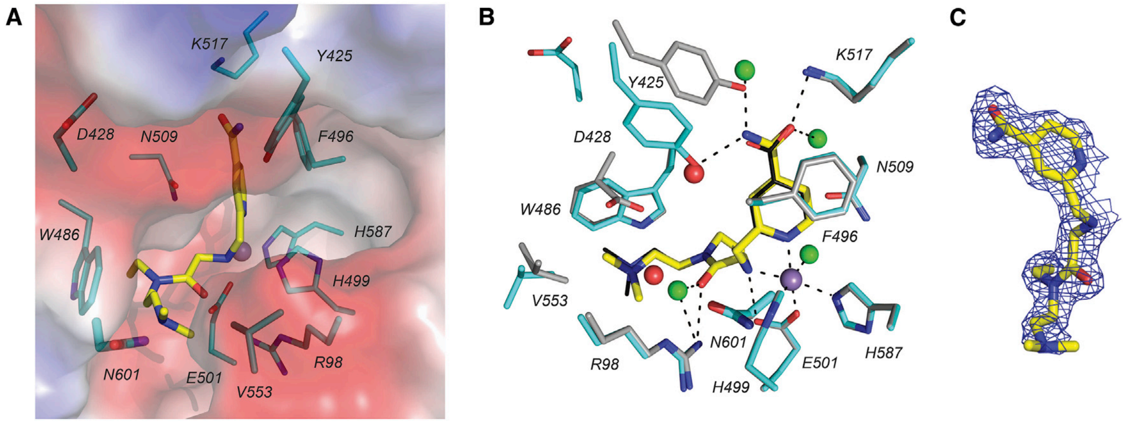

Figure 2. Crystal Structure of KDOAM-25 Bound to KDM5B

(A) Surface representation of KDOAM-25 buried in the 2-OG pocket of KDM5B (PDB: 5A3N).

(B) KDOAM-25 (yellow sticks) binds to KDM5B (cyan sticks) as a bidentate metal chelator. The Mn(II) ion (purple sphere), which serves as the crystallographic surrogate for the catalytic Fe(II), is held in the protein by one Asp and two His residues. Y425 is shifted to accommodate the carboxamide of KDOAM-25 compared with its pose (gray sticks) when binding KDOAM-20 (thin black sticks from PDB: 5A3T), which allows binding of an additional water molecule within the pocket (KDOAM-25, green spheres; KDOAM-20, red spheres).

(C) Electron density (blue mesh from $2F_0-F_c$ map) of KDOAM-25 in the co-crystal structure with KDM5B. See Table S2 for crystallographic statistics.

of ~50 μM (Figure 3A) and the other KDM5 family members at concentrations above 100 μM (Figure 3B), but showed no cellular activity on any of the other tested JmjC family members (Figure 3C). At higher concentrations, all compounds showed increased H3K4me3 levels for both WT and MUT overexpressing cells, indicating that the observed change in histone methylation is likely a combination of inhibition of endogenous KDM5 as well as the overexpressed enzyme, which made determination of an accurate IC_{50} challenging.

KDOAM-25 also inhibited the activity of endogenous KDM5 demethylases with potencies similar to those seen in the overexpression assay (Figure S1C), and was found to be active in increasing H3K4me3 levels, using chromatin immunoprecipitation followed by parallel sequencing (ChIP-seq), at similar concentrations in human multiple myeloma cells (see below).

The exquisite biochemical selectivity profile of KDOAM-25 indicates that the increase in the H3K4me3 mark is due to the inhibition of KDM5 enzymes. The other demethylase capable of removing the H3K4me3 mark is KDM2B. KDM2B is structurally and phylogenetically related to KDM2A (69% sequence similarity), for which KDOAM-25 is a weak inhibitor with an IC_{50} of 4.4 μM (Table S1).

The higher concentration (60 μM) required for cellular activity could be ascribed to different reasons: first to the partial competition with the co-factor 2-OG, the physiological intracellular concentrations of which could be up to micromolar levels (Thirstrup et al., 2011); second to an estimated moderate-to-low cell permeability of KDOAM25. A Caco-2 cell permeability assay was used to evaluate the passive permeation through the cellular membrane (Table S3), and KDOAM25 has shown values in the low permeability region. Together these effects could explain the partial loss of potency observed in the IF cell assay.

Although KDOAM-25 is a potent KDM5 inhibitor in vitro, we could not rule out the possibility that some of the measured cellular affects may be due to amide hydrolysis to give KDOAM-20 in situ. For this reason we investigated the chemical stability of KDOAM-25 in phosphate buffer and its biological stability in liver microsomes (Tables S3 and S4). Incubation of the compound at 37°C in PBS did not show any hydrolysis of the amide after 24 hr, while the ester KDOAM-21 was partly hydrolyzed to the corresponding acid KDOAM-20 in 25% after the same time. Similarly, incubation of KDOAM25 with different liver microsomes (human, mouse, and rat) showed low intrinsic clearance in all species (Table S3), indicating that the compound is likely to have good biological stability within a cellular environment.

Increased *KDM5B* Expression Is Associated with Shorter Survival in Myeloma Patients and Ex Vivo Inhibition with KDOAM-25 Results in Cell-Cycle Arrest

After having identified a selective and cell-active KDM5 inhibitor, we then went on to employ this molecule in ex vivo experiments in MM1S multiple myeloma cells. In line with various reports on the oncogenic roles of the KDM5 enzymes (Kooistra and Helin, 2012), we found that the H3K4me3 demethylase KDM5B is indeed a predictive factor in multiple myeloma. We performed survival analysis using data from three separate, large clinical datasets of newly diagnosed myeloma patients for whom the level of (*KDM5B*) gene expression was measured at diagnosis. Univariate analysis of the data from each trial indicated that higher levels of expression of *KDM5B* were associated with worse overall survival, with significantly shorter survival seen in patients with expression in the upper quartile compared with those having lower expression levels. A further multivariate analysis of the data from the Myeloma IX trial, for which the most complete dataset was available, indicates that the highest quartile of *KDM5B* expression at diagnosis remains associated with a statistically worse outcome compared with lower *KDM5B* expression ($p = 0.039$). These data further highlight the importance of chromatin-modification mechanisms and, in particular, the H3K4me3 demethylase KDM5B as an important factor in multiple myeloma (Figure 4A).

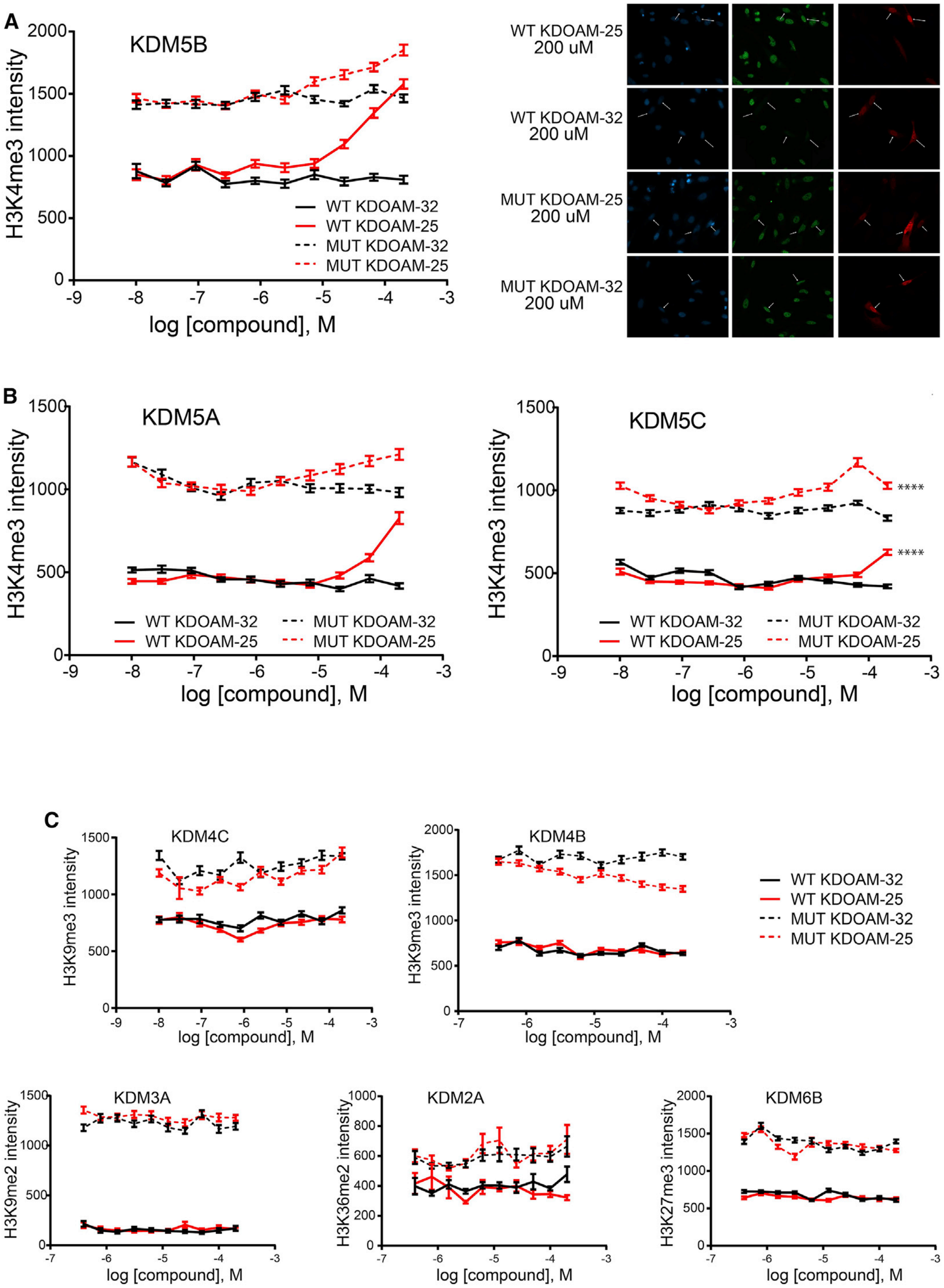

Figure 3. KDOAM-25 Inhibits KDM5 Activity in Cells

(A) Half maximal effective concentration curves for KDOAM-25 against cells overexpressing FLAG-tagged KDM5B (left). Immunofluorescence assay showing inhibition of KDM5B (WT)-mediated H3K4me3 demethylation by KDOAM-25, but no effect of the negative control compound KDOAM-32. Immunofluorescence images showing HeLa cells stained with DAPI nuclear stain (blue), a specific antibody against H3K4me3 (green), and FLAG tag antibody (red) (right). Cells were transfected with KDM5B and treated with the indicated inhibitors. Arrowheads indicate cells transfected with KDM5B-FLAG.

(B) KDM5A and KDM5C are inhibited to a lesser extent by KDOAM-25 in cells overexpressing the respective demethylase. ****p $\leq$ 0.0001.

(C) KDOAM-25 has no effect on the histone substrates of the other KDM family members tested. No effect of any of the compounds is seen on any of the overexpressing catalytically inactive mutants, MUT; dotted lines in (A), (B), and (C). IF was read after 24 hr of compound treatment. Data represents the average and SE of at least 100 cells.

Please cite this article as: Tumber et al., Potent and Selective KDM5 Inhibitor Stops Cellular Demethylation of H3K4me3 at Transcription Start Sites and Proliferation of MM1S Myeloma Cells, Cell Chemical Biology (2017), http://dx.doi.org/10.1016/j.chembiol.2017.02.006

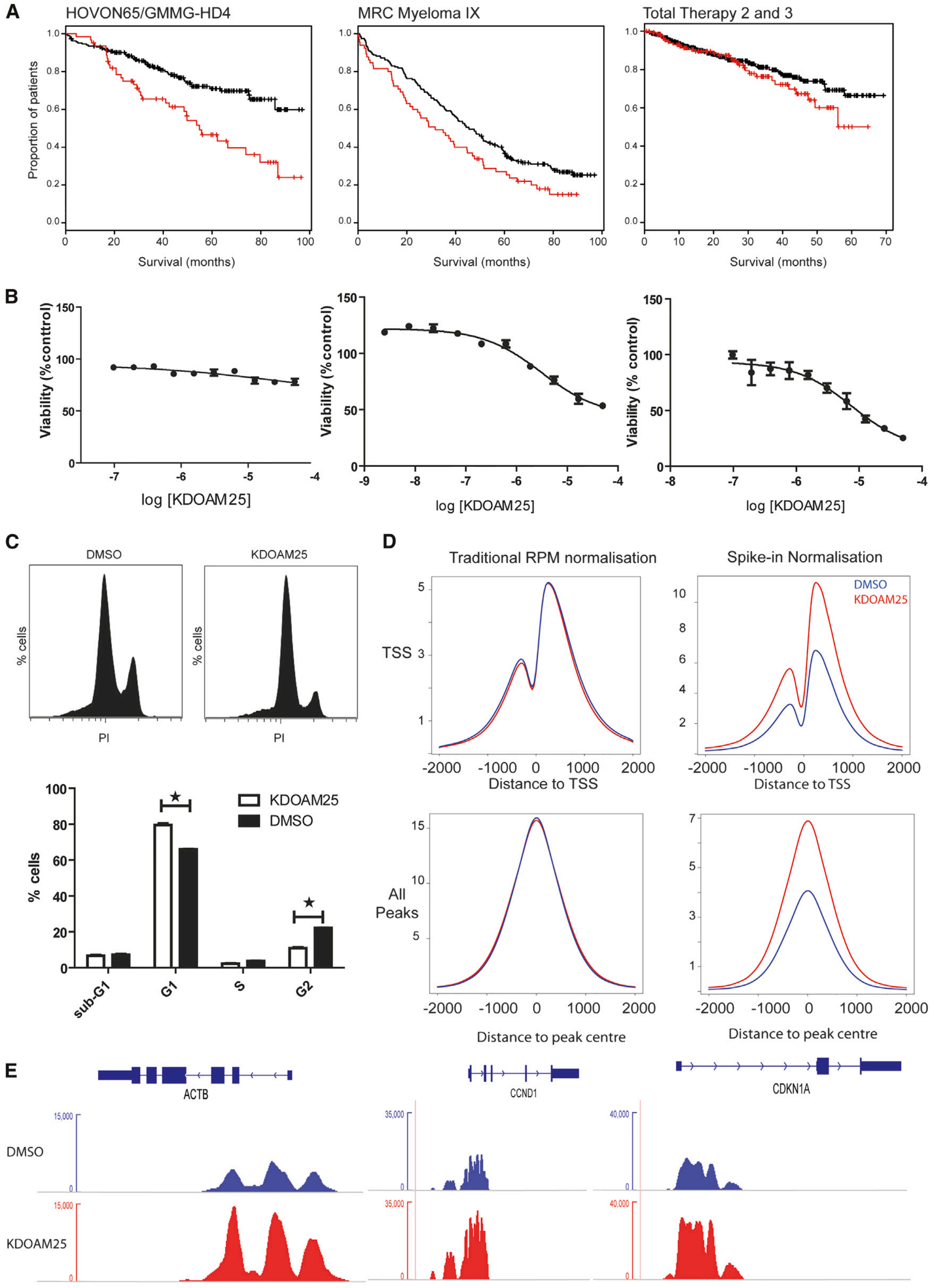

Figure 4. KDM5B and KDOAM-25 in Multiple Myeloma Cells

(A) Increased histone H3K4me3 demethylase *KDM5B* expression is associated with shorter overall survival in multiple myeloma. Data from Affymetrix gene expression analysis with linked survival was available from three large datasets of myeloma patients at diagnosis (Hovon65/GMMG-HD4 trial [n = 246, GEO: GSE19784], MRC Myeloma IX trial [n = 259], Total Therapy 2 and 3 trials [n = 559, GEO: GSE2658]). Results for the probeset 201548_s_at (*KDM5B*) are shown as Kaplan-Meier survival curves for patients comparing those with the highest quartile of expression (red) of KDM5B to all others (black).

(legend continued on next page)

To investigate the role of the inhibition of H3K4 demethylation we screened the anti-proliferative effects of KDOAM-25 in the MM1S multiple myeloma cell line. Using a fluorescent cell-viability assay, we found that after a delay of 5–7 days, KDOAM-25 was able to reduce the viability of MM1S cells with an IC_{50} of ~30 μM with little effect on cell viability after 3 days (Figure 4B). KDOAM-25 treatment did not show the same decrease in viability in a range of other multiple myeloma cells or in a cell line derived from human mesenchymal stem cells (Figure S3). KDOAM-25 treatment resulted in a G1 cell-cycle arrest with an increased proportion of MM1S in G1 ($p = 0.0286$) and a decrease of the proportion of cells in G2 without an increase in the proportion of cells in the apoptotic sub-G1 phase (Figure 4C). ChIP-seq was performed on MM1S cells treated with KDOAM-25 to investigate the change in the distribution of H3K4me3 marks across the genome. When distribution of H3K4me3 was measured following normalization to reads-per-million mapped reads (RPM) there was little difference seen in the coverage of H3K4me3 at either transcription start sites or across the totality of all peaks called. We then employed the ChIP-Rx strategy to enable quantification of the amount of pulled-down chromatin (Orlando et al., 2014). Use of this spike-in quantification revealed a global change in the level of H3K4me3, with approximately twice as much H3K4me3 found in cells treated with KDOAM-25 compared with the vehicle control (Figure 4D). As the increase in H3K4me3 is global it is also observed at the transcription start site of genes associated with endogenous "housekeeping" within the cell, such as β-actin (ACTB), pro-proliferative genes such as cyclin D1 (CCND1), and anti-proliferative genes such as cyclin-dependent kinase inhibitor 1a (CDKN1A) (Figure 4E).

SIGNIFICANCE

KDOAM-25 is a highly selective inhibitor of the KDM5 sub-family of histone lysine demethylases with strongest activity found against the catalytic domain of KDM5B. KDOAM-25 shows potent inhibition of the KDM5A-D enzymes in vitro (<100 nM), and an expected corresponding increase in H3K4me3 levels using IF detection in an ectopic expression system in HeLa cells was seen with compound concentrations in the two-digit micromolar range. Structure-based design was used to generate KDOAM-25 without the need for the previously reported ester pro-drugs. KDOAM-25 is devoid of off-target activity on a CEREP express panel; it is well tolerated in several cell lines, even at high concentrations. Despite the fact that the compound cannot be considered as a chemical probe according to the SGC criteria (cellular EC_{50} of 1 μM), due to its good stability, high selectivity, and low cytotoxicity KDOAM-25 may be a useful tool, although results should be considered carefully due to the higher cellular EC_{50} of ~50 μM. While there is no inhibition of any other KDM sub-families at this concentration, other off-targets cannot be completely ruled out. Hence, KDOAM-25 could help to investigate the biological roles of the KDM5 enzymes in relevant cellular contexts. For example, here we reported that KDOAM-25 shows an inverse relationship between KDM5B expression and overall survival using data from three clinical multiple myeloma trials, thus suggesting a potential application for KDM5B inhibitors in myeloma therapy. When human myeloma MM1S cells were treated with KDOAM-25 at concentrations consistent with inhibition of KDM5B-mediated H3K4me3 demethylation in myeloma cells, anti-proliferative effects were observed with anticipated genome-wide increases in H3K4me3 levels as demonstrated by quantitative ChIP-seq experiments.

EXPERIMENTAL PROCEDURES

Compound Synthesis

KDOAM-20 (KDM5-C49) and KDOAM-21 (KDM5-C70) were purchased from Xcessbio Biosciences.

KDOAM-25, -28, and -29 were prepared from KDOAM-21 via hydrolysis and amide coupling. KDOAM-32 was prepared from methyl 5-(bromomethyl)-nicotinate. KDOAM-1 was prepared from diethyl pyridine-2,4-dicarboxylate. See the Supplemental Information for full synthetic protocols.

Engineering of KDM5B Constructs for In Vitro Analysis

A KDM5B construct with selective deletion of the PHD and ARID domains, was amplified from an OriGene cDNA clone, and cloned into a pFastBac-derived vector (pFB-LIC-Bse), which contained a tobacco etch virus (TEV) protease-cleavable N-terminal 6x-histidine tag. Successful cloning and correct sequence of this construct were confirmed by Sanger sequencing.

Protein Expression and Purification

A KDM5B construct encoding regions Phe26-Ile770 was amplified from an OriGene cDNA clone and cloned into a pFastBac-derived vector (pFB-LIC-Bse) containing a TEV protease-cleavable N-terminal 6x-histidine tag. The recombinant KDM5B (residues 26–770) construct was expressed in Sf9 cells, and generation of recombinant baculoviruses, insect cell culture, and infections was performed according to the manufacturer's instructions (Invitrogen) (Johansson et al., 2016). The cells were harvested 72 hr post infection and lysed in a buffer containing 50 mM HEPES (pH 7.5), 500 mM NaCl, 10 mM imidazole, 5% glycerol, 0.5 mM Tris(2-carboxyethy1)phosphine, and a proteinase inhibitor cocktail (Calbiochem), and purified using nickel-affinity chromatography using a stepwise gradient of imidazole. The eluted protein was then incubated with TEV protease at 4°C overnight, followed by size-exclusion chromatography (Superdex 200). The TEV protease and uncleaved protein were removed using nickel-affinity chromatography and protein was concentrated to 8.1 mg/mL and stored at −80°C.

Protein Crystallization, X-Ray Data Collection, and Structure Determination

Protein preparations were concentrated in Amicon concentrators to 8.1 mg/mL, and were subjected to crystallization experiments at 4°C using the sitting-drop vapor diffusion method. The protein was pre-incubated with 1 mM KDOAM-25 and 4 mM $MnCl_2$ before the protein-compound mixture

(B) Viability of MM1S when treated with KDOAM-25 as measured after 3, 5, or 7 days (mean ± SE of three independent experiments, each in quadruplicate and normalized to DMSO control).

(C) KDOAM-25 significantly increases the proportion of cells in G1 and reduces the proportion in G2 without increasing the sub-G1 population. *$p < 0.05$.

(D) Average coverage of H3K4me3 marks across transcription start sites (top) and across all called-peaks (bottom) when normalized by either reads per million (left) or spike-in quantification (right) (mean of three independent experiments). KDOAM-25 data shown in red and DMSO in blue.

(E) Coverage tracks of H3K4me3 normalized by spike-in quantification for three genes, a "housekeeping" gene *ACTB*, a pro-proliferative gene *cyclin D1* and an anti-proliferative gene *CDKN1A*.

was transferred to crystallization plates. KDM5B yielded diffracting crystals with KDOAM-25 in a drop consisting of 50 nL protein-compound mix (8.1 mg/mL) and 100 nL of a precipitant consisting of 1.6 M Na/K phosphate, 0.1 M HEPES (pH 7.5), and 20 nL of KDM5B seeds of crystals obtained from the same condition. Crystal was cryoprotected with mother liquor supplemented with 25% ethylene glycol before they were flash frozen in liquid nitrogen. The dataset was collected on beamline I02 at the Diamond Light Source. The crystal belongs to P6522 diffracted to 2.0 Å, and the dataset was processed with XDS and scaled and merged with Aimless. The structure was refined with a KDM5B model (51af). The protein-ligand model was further improved by subsequent cycles of model building in Coot and refinement with Phenix. The ligand library was generated with ELBOW. The quality of the structure was assessed with MolProbity server.

Determination of Cellular H3K4me3 Levels

The human cervical carcinoma cell line HeLa was obtained from the American Type Cultures Collection and maintained in Eagle's minimal essential media (Sigma-Aldrich) supplemented with 10% heat-inactivated fetal bovine serum (Life Technologies). Full-length cDNA for KDM5B (JARID1B, Q9UGL1, cDNA), KDM5C (JARID1C, P41229, IMAGE 5492114), KDM4B (JMJD2B, O94953, gift from ICR), KDM4C (JMJD2C, Q9H3R0, 8143862), KDM3A (JMJD1A, Q9Y4C1, 4823253), KDM2A (FBXL11, Q9Y2K7, 5534384), and KDM6B (JMJD3, O15054, cDNA) were amplified by PCR from either an MGC clone or a commercial cDNA source, or received as a gift from collaborators and cloned into pDONR-221 vector using Gateway BP reaction producing Gateway entry clones.

To produce catalytically inactive KDMs, residues involved in iron coordination were mutated to alanine (KDM5B H499A/E501A, KDM5C H513A/E515A, KDM4B H189A/E191A, KDM4C H190A/E192A, KDM3A H1120Y, KDM2A H212A/D214A, and KDM6B H1390/E1392). Mutations were introduced into full-length KDM Gateway entry clones using 15 cycles of the QuikChange II PCR protocol (Agilent Technologies). Mammalian expression constructs encoding N-terminal 3*FLAG were constructed using the Gateway LR recombination reaction between pCDNA5-FRT/TO-3FLAG destination vector (Lambert et al., 2014) and the WT or mutated KDM Gateway entry clone. Experimental details describing the overexpression KDM IF assay have been published previously (Bavetsias et al., 2016; Hopkinson et al., 2013).

In brief, cells were transiently transfected with either the FLAG-tagged WT or mutant demethylase described above, using Lipofectamine 2000 (Life Technologies). Four hours after transfection the cells were treated with serial dilutions of compound for 24 hr. The cells were subsequently fixed and stained with an anti-FLAG antibody (Sigma F3165) as well as an anti-methylated histone antibody (H3K4me3 Diagenode C15410003, H3K9me2 Abcam ab1220, H3K9me3 Abcam ab8898, H3K27me3 Millipore 07–449, or H3K36me2 Active Motif 61019). The secondary antibodies were labeled with either Alexa Fluor 488 or 568 (Life Technologies). Image acquisition was conducted using the Operetta High Content Imaging System (PerkinElmer) and image analysis was performed with the Columbus software (PerkinElmer). After the DAPI-stained nuclei were automatically identified, a minimum threshold for anti-FLAG intensity was set so that only cells highly expressing the demethylase were analyzed. Assays assessing the endogenous H3K4me3 methyl mark changes were performed accordingly without overexpressing KDM5 after compounds treatment for 48 hr.

Sf9 Genome Sequencing

Sf9 cells, derived from the fall armyworm (*Spodoptera frugiperda*), routinely used for protein expression within the laboratory, were used. The genomic DNA from 10^7 cells was extracted using the Quick-gDNA MiniPrep Kit (Zymo Research) according to the manufacturer's instruction and sonicated to an average size of approximately 350 bp. Illumina-compatible paired-end libraries were constructed using the NebNext Ultra DNA Kit (NEB) and sequenced on a NextSeq500 (Illumina) with 83 bp reads in both directions. One hundred million pairs of reads were error corrected using Fiona (Schulz et al., 2014) prior to de novo genome assembly using Spades (Bankevich et al., 2012). This process was repeated using a separate 100 million pair read, and a consensus assembly between the two de novo assemblies was created using AMOS and MUMmer. For use in quantitative ChIP, the assembled contigs were concatenated to the human genome (hg38) to generate a merged genome, and indexes for aligning were built with Bowtie.

ChIP-Seq

MM1S cells (10^7) were treated with either 50 μM KDOAM-25 or DMSO for 7 days. Immediately prior to fixation in 1% formaldehyde, three million sf9 cells were added to each sample. Cells were fixed for 8 min at room temperature, lysed, and sonicated on a Bioruptor Pico for 3 repeats of 10 cycles of 30 s on/30 s off. The sonicated lysates were incubated for 1 hr at 4°C with pre-blocked protein A Sepharose beads, prior to overnight incubation with 1 μg of anti-H3K4me3 antibody (Millipore cat no. 07–473 lot no. 2207281). Pre-blocked Sepharose beads were added for 1 hr, the beads washed, and DNA was eluted followed by Proteinase K digestion. Libraries were constructed using the NebNext Ultra DAN Kit (NEB) and sequenced on an Illumina NextSeq 500 platform. Libraries were also generated for the input of each condition and sequenced in a similar manner.

Data Processing of ChIP-Seq

Sequencing reads were aligned to the concatenated human/sf9 genome generated earlier using Bowtie (version 1.1.2), allowing only reads that matched uniquely to one location. The number of reads mapping to the human and sf9 genomes separately in each condition was calculated. The number of sf9 reads-per-million total aligned reads was calculated for each pull-down condition and corrected according to the percentage of sf9 reads in the corresponding input samples. A scaling factor for normalization was then calculated for each pull-down based on the reciprocal of the corrected sf9 reads per million for each sample.

The aligned Bam files were converted to bedGraph files using BedTools for each strand of each sample using the scaling factor derived earlier. Homer v4.8 was used to read the produced bedGraphs for each sample and create a Tag Directory for each sample that was used for subsequent calculations of coverage. Peaks were called using macs2 with the "–broad option" and the gapped-peaks called used for downstream analysis.

Coverage at the transcription start site and across each peak was calculated for each condition using Homer for both reads normalized on a reads-per-million basis and those normalized with the Sf9 spike in and an average taken to produce the values plotted.

Measurement of Cell Viability

MM1S myeloma cells were cultured in RMPI with 10% heat-inactivated FCS and 2 mM glutamine under standard conditions. A total of 5×10^5 cells per well was seeded into a 96-well plate and treated with a range of doses of KDOAM-25: from 50 mM to approximately 10 nM for 7 days. Viability was measured using PrestoBlue reagent and normalized to a vehicle control. Data were plotted and IC_{50} values were calculated in Prism (v5.1).

Survival Curves from Clinical Trials

Gene expression profiling was performed on the CD138-selected plasma cells from newly diagnosed multiple myeloma patients. Data from expression analysis with linked survival was available for three large trials of myeloma patients at diagnosis. These are the MRC Myeloma IX trial (n = 259), the Hovon65/GMMG-HD4 trial (n = 246), and the Total Therapy 2 and 3 trials (n = 559). Results for the Affymetrix probe 201548_s_at were used. Kaplan-Meier survival curves for patients comparing high expressors of KDM5B with all others were plotted.

SUPPLEMENTAL INFORMATION

Supplemental Information includes Supplemental Experimental Procedures, three figures, and five tables and can be found with this article online at http://dx.doi.org/10.1016/j.chembiol.2017.02.006.

AUTHOR CONTRIBUTIONS

Conceptualization, Methodology, Writing, Visualization, P.B., U.O., and S. Müller; Investigation, Formal Analysis, A.K., A.N., A.S., A.T., C.B., C.J., C.P., C. Schofield, C. Strain-Damerell, C.Y., E.H., F.D., G.M., G.R., K.E., N.A., N.B., N.L., N.W., O.F., P.B., P.S., R.N., S.H., S. Munro., S. Müller, S.V.,

and U.O.; Supervision, C. Schofield., N.L., P.B., U.O., and S. Müller; Funding Acquisition, C.B., N.A., P.B., U.O., and S. Müller.

ACKNOWLEDGMENTS

The SGC is a registered charity (no. 1097737) that receives funds from AbbVie, Bayer Pharma AG, Boehringer Ingelheim, Canada Foundation for Innovation, Eshelman Institute for Innovation, Genome Canada, Innovative Medicines Initiative (EU/EFPIA) (ULTRA-DD grant no. 115766), Janssen, Merck, Novartis Pharma AG, Ontario Ministry of Economic Development and Innovation, Pfizer, São Paulo Research Foundation-FAPESP, Takeda, and Wellcome Trust (092809/Z/10/Z). Research in our laboratories is further supported through funding from Arthritis Research UK (program grant number 20522), the NIHR Oxford Biomedical Research Unit, Bone Cancer Research UK, and the Rosetrees Trust. C.J.S. is funded by CRUK (C8717/A18245). A.K. is a Royal Society Dorothy Hodgkin Research Fellow. We would like to thank Professor Peter Sonneveld and Professor Hartmut Goldschmidt for the data from the HOVON/GMMG-HD4 trial.

Received: April 2, 2016
Revised: October 31, 2016
Accepted: February 1, 2017
Published: March 2, 2017

REFERENCES

Akimoto, C., Kitagawa, H., Matsumoto, T., and Kato, S. (2008). Spermatogenesis-specific association of SMCY and MSH5. Genes to Cells *13*, 623–633.

Bankevich, A., Nurk, S., Antipov, D., Gurevich, A.A., Dvorkin, M., Kulikov, A.S., Lesin, V.M., Nikolenko, S.I., Pham, S., Prjibelski, A.D., et al. (2012). SPAdes: a new genome assembly algorithm and its applications to single-cell sequencing. J. Comput. Biol. *19*, 455–477.

Barrett, A., Madsen, B., Copier, J., Lu, P.J., Cooper, L., Scibetta, A.G., Burchell, J., and Taylor-Papadimitriou, J. (2002). PLU-1 nuclear protein, which is upregulated in breast cancer, shows restricted expression in normal human adult tissues: a new cancer/testis antigen? Int. J. Cancer *101*, 581–588.

Bavetsias, V., Lanigan, R.M., Ruda, G.F., Atrash, B., McLaughlin, M.G., Tumber, A., Mok, N.Y., Le Bihan, Y.-V., Dempster, S., Boxall, K.J., et al. (2016). 8-Substituted pyrido[3,4-d]pyrimidin-4(3H)-one derivatives as potent, cell permeable, KDM4 (JMJD2) and KDM5 (JARID1) histone lysine demethylase inhibitors. J. Med. Chem. *59*, 1388–1409.

Cellot, S., Hope, K.J., Chagraoui, J., Sauvageau, M., Deneault, E., MacRae, T., Mayotte, N., Wilhelm, B.T., Landry, J.R., Ting, S.B., et al. (2013). RNAi screen identifies Jarid1b as a major regulator of mouse HSC activity. Blood *122*, 1545–1555.

Chai, X., Nagarajan, S., Kim, K., Lee, K., and Choi, J.K. (2013). Regulation of the boundaries of accessible chromatin. PLoS Genet. *9*, e1003778.

Dey, B.K., Stalker, L., Schnerch, A., Bhatia, M., Taylor-Papidimitriou, J., and Wynder, C. (2008). The histone demethylase KDM5b/JARID1b plays a role in cell fate decisions by blocking terminal differentiation. Mol. Cell Biol. *28*, 5312–5327.

ENCODE Project Consortium. (2012). An integrated encyclopedia of DNA elements in the human genome. Nature *489*, 57–74.

England, K.S., Tumber, A., Krojer, T., Scozzafava, G., Ng, S.S., Daniel, M., Szykowska, A., Che, K., von Delft, F., Burgess-Brown, N.A., et al. (2014). Optimisation of a triazolopyridine based histone demethylase inhibitor yields a potent and selective KDM2A (FBXL11) inhibitor. MedChemComm *5*, 1879–1886.

Gehling, V.S., Bellon, S.F., Harmange, J.-C., LeBlanc, Y., Poy, F., Odate, S., Buker, S., Lan, F., Arora, S., Williamson, K.E., et al. (2016). Identification of potent, selective KDM5 inhibitors. Bioorg. Med. Chem. Lett. *26*, 4350–4354.

Hopkinson, R.J., Tumber, A., Yapp, C., Chowdhury, R., Aik, W., Che, K.H., Li, X.S., Kristensen, J.B.L., King, O.N.F., Chan, M.C., et al. (2013). 5-Carboxy-8-hydroxyquinoline is a broad spectrum 2-oxoglutarate oxygenase inhibitor which causes iron translocation. Chem. Sci. *4*, 3110–3117.

Horton, J.R., Liu, X., Gale, M., Wu, L., Shanks, J.R., Zhang, X., Webber, P.J., Bell, J.S., Kales, S.C., Mott, B.T., et al. (2016). Structural basis for KDM5A histone lysine demethylase inhibition by diverse compounds. Cell Chem. Biol. *23*, 769–781.

Itoh, Y., Sawada, H., Suzuki, M., Tojo, T., Sasaki, R., Hasegawa, M., Mizukami, T., and Suzuki, T. (2015). Identification of Jumonji AT-rich interactive domain 1A inhibitors and their effect on cancer cells. ACS Med. Chem. Lett. *6*, 665–670.

Johansson, C., Tumber, A., Che, K., Cain, P., Nowak, R., Gileadi, C., and Oppermann, U. (2014). The roles of Jumonji-type oxygenases in human disease. Epigenomics *6*, 89–120.

Johansson, C., Velupillai, S., Tumber, A., Szykowska, A., Hookway, E.S., Nowak, R.P., Strain-Damerell, C., Gileadi, C., Philpott, M., Burgess-Brown, N., et al. (2016). Structural analysis of human KDM5B guides histone demethylase inhibitor development. Nat. Chem. Biol. *12*, 539–545.

Kooistra, S.M., and Helin, K. (2012). Molecular mechanisms and potential functions of histone demethylases. Nat. Rev. Mol. Cell Biol. *13*, 297–311.

Kruidenier, L., Chung, C.-w., Cheng, Z., Liddle, J., Che, K., Joberty, G., Bantscheff, M., Bountra, C., Bridges, A., Diallo, H., et al. (2012). A selective Jumonji H3K27 demethylase inhibitor modulates the proinflammatory macrophage response. Nature *488*, 404–408.

Labelle, M., Boesen, T., Mehrotra, M., Khan, Q., and Ullah, F. (2014). Inhibitors of histone demethylases, Patent WO2014053491 A1, filed October 1, 2013, and published April 10, 2014.

Lambert, J.P., Tucholska, M., Pawson, T., and Gingras, A.C. (2014). Incorporating DNA shearing in standard affinity purification allows simultaneous identification of both soluble and chromatin-bound interaction partners. J. Proteomics *100*, 55–59.

Lauberth, S.M., Nakayama, T., Wu, X., Ferris, A.L., Tang, Z., Hughes, S.H., and Roeder, R.G. (2013). H3K4me3 interactions with TAF3 regulate preinitiation complex assembly and selective gene activation. Cell *152*, 1021–1036.

Li, L., Wang, L., Song, P., Geng, X., Liang, X., Zhou, M., Wang, Y., Chen, C., Jia, J., and Zeng, J. (2014). Critical role of histone demethylase RBP2 in human gastric cancer angiogenesis. Mol. Cancer *13*, 1–13.

Liang, X., Zeng, J., Wang, L., Fang, M., Wang, Q., Zhao, M., Xu, X., Liu, Z., Li, W., Liu, S., et al. (2013). Histone demethylase retinoblastoma binding protein 2 is overexpressed in hepatocellular carcinoma and negatively regulated by hsa-miR-212. PLoS One *8*, e69784.

Liang, J., Zhang, B., Labadie, S., Ortwine, D.F., Vinogradova, M., Kiefer, J.R., Gehling, V.S., Harmange, J.-C., Cummings, R., Lai, T., et al. (2016). Lead optimization of a pyrazolo[1,5-a]pyrimidin-7(4H)-one scaffold to identify potent, selective and orally bioavailable KDM5 inhibitors suitable for in vivo biological studies. Bioorg. Med. Chem. Lett. *26*, 4036–4041.

Madsen, B., Tarsounas, M., Burchell, J.M., Hall, D., Poulsom, R., and Taylor-Papadimitriou, J. (2003). PLU-1, a transcriptional repressor and putative testis-cancer antigen, has a specific expression and localisation pattern during meiosis. Chromosoma *112*, 124–132.

McAllister, T.E., England, K.S., Hopkinson, R.J., Brennan, P.E., Kawamura, A., and Schofield, C.J. (2016). Recent progress in histone demethylase inhibitors. J. Med. Chem. *59*, 1308–1329.

Ng, S.S., Yue, W.W., Oppermann, U., and Klose, R.J. (2009). Dynamic protein methylation in chromatin biology. Cell Mol. Life Sci. *66*, 407–422.

Orlando, D.A., Chen, M.W., Brown, V.E., Solanki, S., Choi, Y.J., Olson, E.R., Fritz, C.C., Bradner, J.E., and Guenther, M.G. (2014). Quantitative ChIP-seq normalization reveals global modulation of the epigenome. Cell Rep. *9*, 1163–1170.

Pasini, D., Hansen, K.H., Christensen, J., Agger, K., Cloos, P.A., and Helin, K. (2008). Coordinated regulation of transcriptional repression by the RBP2 H3K4 demethylase and Polycomb-Repressive Complex 2. Genes Dev. *22*, 1345–1355.

Roesch, A., Fukunaga-Kalabis, M., Schmidt, E.C., Zabierowski, S.E., Brafford, P.A., Vultur, A., Basu, D., Gimotty, P., Vogt, T., and Herlyn, M. (2010). A

temporarily distinct subpopulation of slow-cycling melanoma cells is required for continuous tumor growth. Cell *141*, 583–594.

Santos-Reboucas, C.B., Fintelman-Rodrigues, N., Jensen, L.R., Kuss, A.W., Ribeiro, M.G., Campos, M., Jr., Santos, J.M., and Pimentel, M.M. (2011). A novel nonsense mutation in KDM5C/JARID1C gene causing intellectual disability, short stature and speech delay. Neurosci. Lett. *498*, 67–71.

Schiller, R., Scozzafava, G., Tumber, A., Wickens, J.R., Bush, J.T., Rai, G., Lejeune, C., Choi, H., Yeh, T.L., Chan, M.C., et al. (2014). A cell-permeable ester derivative of the JmjC histone demethylase inhibitor IOX1. ChemMedChem *9*, 566–571.

Schulz, M.H., Weese, D., Holtgrewe, M., Dimitrova, V., Niu, S., Reinert, K., and Richard, H. (2014). Fiona: a parallel and automatic strategy for read error correction. Bioinformatics (Oxford, England) *30*, i356–i363.

Tanny, J.C. (2014). Chromatin modification by the RNA polymerase II elongation complex. Transcription *5*, e988093.

Teng, Y.C., Lee, C.F., Li, Y.S., Chen, Y.R., Hsiao, P.W., Chan, M.Y., Lin, F.M., Huang, H.D., Chen, Y.T., Jeng, Y.M., et al. (2013). Histone demethylase RBP2 promotes lung tumorigenesis and cancer metastasis. Cancer Res. *73*, 4711–4721.

Thirstrup, K., Christensen, S., Møller, H.A., Ritzén, A., Bergström, A.-L., Sager, T.N., and Jensen, H.S. (2011). Endogenous 2-oxoglutarate levels impact potencies of competitive HIF prolyl hydroxylase inhibitors. Pharmacol. Res. *64*, 268–273.

Vinogradova, M., Gehling, V.S., Gustafson, A., Arora, S., Tindell, C.A., Wilson, C., Williamson, K.E., Guler, G.D., Gangurde, P., Manieri, W., et al. (2016). An inhibitor of KDM5 demethylases reduces survival of drug-tolerant cancer cells. Nat. Chem. Biol. *12*, 531–538.

Wang, G.G., Song, J., Wang, Z., Dormann, H.L., Casadio, F., Li, H., Luo, J.L., Patel, D.J., and Allis, C.D. (2009). Haematopoietic malignancies caused by dysregulation of a chromatin-binding PHD finger. Nature *459*, 847–851.

Wang, L., Chang, J., Varghese, D., Dellinger, M., Kumar, S., Best, A.M., Ruiz, J., Bruick, R., Peña-Llopis, S., Xu, J., et al. (2013). A small molecule modulates Jumonji histone demethylase activity and selectively inhibits cancer growth. Nat. Commun. *4*, 2035.

Wang, L., Mao, Y., Du, G., He, C., and Han, S. (2015). Overexpression of JARID1B is associated with poor prognosis and chemotherapy resistance in epithelial ovarian cancer. Tumour Biol. *36*, 2465–2472.

Westaway, S.M., Preston, A.G.S., Barker, M.D., Brown, F., Brown, J.A., Campbell, M., Chung, C.-w., Diallo, H., Douault, C., Drewes, G., et al. (2016a). Cell penetrant inhibitors of the KDM4 and KDM5 families of histone lysine demethylases. 1. 3-Amino-4-pyridine carboxylate derivatives. J. Med. Chem. *59*, 1357–1369.

Westaway, S.M., Preston, A.G.S., Barker, M.D., Brown, F., Brown, J.A., Campbell, M., Chung, C.-w., Drewes, G., Eagle, R., Garton, N., et al. (2016b). Cell penetrant inhibitors of the KDM4 and KDM5 families of histone lysine demethylases. 2. Pyrido[3,4-d]pyrimidin-4(3H)-one derivatives. J. Med. Chem. *59*, 1370–1387.

Wu, X., Fang, Z., Yang, B., Zhong, L., Yang, Q., Zhang, C., Huang, S., Xiang, R., Suzuki, T., Li, L.-L., et al. (2016). Discovery of KDM5A inhibitors: homology modeling, virtual screening and structure–activity relationship analysis. Bioorg. Med. Chem. Lett. *26*, 2284–2288.

Zeng, J., Ge, Z., Wang, L., Li, Q., Wang, N., Björkholm, M., Jia, J., and Xu, D. (2010). The histone demethylase RBP2 Is overexpressed in gastric cancer and its inhibition triggers senescence of cancer cells. Gastroenterology *138*, 981–992.

Cell Chemical Biology, Volume *24*

Supplemental Information

Potent and Selective KDM5 Inhibitor Stops Cellular Demethylation of H3K4me3 at Transcription Start Sites and Proliferation of MM1S Myeloma Cells

Anthony Tumber, Andrea Nuzzi, Edward S. Hookway, Stephanie B. Hatch, Srikannathasan Velupillai, Catrine Johansson, Akane Kawamura, Pavel Savitsky, Clarence Yapp, Aleksandra Szykowska, Na Wu, Chas Bountra, Claire Strain-Damerell, Nicola A. Burgess-Brown, Gian Filippo Ruda, Oleg Fedorov, Shonagh Munro, Katherine S. England, Radoslaw P. Nowak, Christopher J. Schofield, Nicholas B. La Thangue, Charlotte Pawlyn, Faith Davies, Gareth Morgan, Nick Athanasou, Susanne Müller, Udo Oppermann, and Paul E. Brennan

Supplemental Information for

A Selective KDM5 Inhibitor, KDOAM-25, inhibits proliferation of MM1S myeloma cells in vitro.

Table S1 related to Figure 1 2-Oxoglutarate Oxygenase Assay Panel

pIC_{50}*	KDOAM-1	KDOAM-20	KDOAM-21	KDOAM-28	KDOAM-29	KDOAM-25	KDOAM-32
**KDM5**‡							
A	*ND*	8.0 ± 0.11 (4)	7.2 ± 0.046 (2)	6.6 ± 0.11 (2)	6.5 ± 0.12 (2)	7.1 ± 0.11 (32)	
B	*ND*	8.2 ± 0.14 (8)	7.3 ± 0.16 (4)	7.0 ± 0.092 (2)	6.6 ± 0.098 (2)	7.7 ± 0.18 (50)	< 4 (2)
C	6.0 ± 0.055 (2)	7.7 ± 0.10 (8)	6.7 ± 0.17 (4)	6.8 ± 0.096 (2)	6.1 ± 0.16 (2)	7.2 ± 0.16 (32)	
D	*ND*	7.7 ± 0.070 (4)	7.0 ± 0.044 (2)	6.8 ± 0.10 (2)	5.9 ± 0.087 (2)	7.2 ± 0.18 (34)	
**KDM3**‡							
A	< 4 (2)	6.0 ± 0.19 (4)	5.6 ± 0.14 (2)	*ND*	*ND*	4.5 ± 0.44 (20)	
B	*ND*	*ND*	*ND*	*ND*	*ND*	4.1 ± 0.65 (8)	
**KDM4**‡							
A	*ND*	6.2 ± 0.10 (2)	5.0 ± 0.13 (2)	*ND*	*ND*	5.0 ± 0.69 (6)	
C	5.9 ± 0.63 (2)	6.8 ± 0.14 (6)	5.6 ± 0.21 (4)	*ND*	*ND*	5.3 ± 0.28 (12)	
E	5.0 ± 0.16 (2)	*ND*	*ND*	*ND*	*ND*	*ND*	
**KDM6B**‡	< 4 (2)	5.9 ± 0.10 (2)	5.0 ± 0.13 (2)	5.1 ± 0.14 (2)	ND	4.0 ± 1.1 (6)	
**KDM2A**‡	4.3 ± 0.50 (2)	5.7 ± 0.050 (2)	4.7 ± 0.15 (2)	*ND*	*ND*	4.4 ± 0.86 (4)	
**PHF8**‡	*ND*	*ND*	*ND*	*ND*	*ND*	5.0 ± 0.057 (2)	
**MINA53**#						< 4 (2)	
**NO66**#						< 4 (2)	
**FIH**§						< 4 (2)	
**EGLN1**§						< 4 (2)	

* pIC50 values ± SEM (number of independent values); ‡ AlphaScreen assay; # RapidFire MS assay; § MALDI-TOF assay.

Table S2 related to Figure 2 Crystallographic Statistics for the Structure of the KDM5B/KDOAM-25 Complex

	KDM5B KDOAM-25 (5a3n)
**Data collection**	
Space group	$P6_522$
Cell dimensions	
a, *b*, *c* (Å)	141.87, 141.87, 151.86
α, β, γ (°)	90, 90, 120
Resolution (Å)	19.81 - 2.0
	(2.07 - 2.00)
R_{merge}	0.08047 (0.6908)
$I / \sigma I$	22.81 (2.99)
Completeness (%)	99.29 (93.57)
Redundancy	12.6 (8.4)
**Refinement**	
Resolution (Å)	2.00
No. reflections	763080 (47111)
R_{work} / R_{free}	0.1803/0.2353
No. atoms	
Protein	3671
Ligand/ion	83
Water	238
B-factors	
Protein	36.60
Ligand/ion	43.50
Water	43.30
R.m.s deviations	
Bond lengths (Å)	0.008
Bond angles (°)	1.15

Data were collected from one crystal for each structure.*Values in parentheses are for highest-resolution shell.

Table S3 related to Figure 1 Caco-2 cell permeability assay and Liver microsomes stability of KDOAM-20, -21 and -25

	KDOAM-25	KDOAM-21	KDOAM-20
**Caco-2 A-B**	<0.76	5.3	0.76
**Caco-2 B-A**	<0.76	15	0.25
**PAMPA pH 5**	<0.3	<0.2	<0.13
**PAMPA pH 6.5**	<0.23	0.233	<0.20
**PAMPA pH 7.4**	<0.23	<0.4	<0.20
**HLM Clint**	*11.9 µL/min/mg*	*ND*	*ND*
**MLM Clint**	*8.42 µL/min/mg*	*ND*	*ND*
**RLM Clint**	*<1 µL/min/mg*	*ND*	*ND*

Table S4 related to Figure 1 KDOAM-21 and -25 chemical stability in Phosphate Buffer Saline

	**KDOAM-21**		**KDOAM-25**	
	Relative % at 254 nm		Relative % at 254 nm	
Time	Ester	Acid	Amide	Acid
0 min	100	0	100	0
20 min	100	0	100	0
1 hour	100	0	100	0
4 hours	93	7	100	0
24 hours	75	25	100	0

Table S5 related to Figure 1 KDOAM-25 Activity in the Cerep Express Panel

Assay	% Inhibition of reference (10 µM)	Reference Compound	K_i Ref (nM)
A1 (h) (antagonist radioligand)	-3	DPCPX	0.56
A2A (h) (agonist radioligand)	-2	NECA	21
A3 (h) (agonist radioligand)	-6	IB-MECA	0.19
alpha 1 (non-selective) (antagonist radioligand)	-15	prazosin	0.1
alpha 2 (non-selective) (antagonist radioligand)	-11	yohimbine	37
beta 1 (h) (agonist radioligand)	5	atenolol	230
beta 2 (h) (agonist radioligand)	5	ICI 118551	0.17
AT1 (h) (antagonist radioligand)	-7	saralasin	0.46
BZD (central) (agonist radioligand)	-42	diazepam	5.9
B2 (h) (agonist radioligand)	-10	NPC 567	9
CB1 (h) (agonist radioligand)	-1	CP 55940	1.3
CCK1 (CCKA) (h) (agonist radioligand)	-3	CCK-8s	0.22
D1 (h) (antagonist radioligand)	10	SCH 23390	0.19
D2S (h) (antagonist radioligand)	19	(+)butaclamol	0.73
ETA (h) (agonist radioligand)	-6	endothelin-1	0.015
GABA (non-selective) (agonist radioligand)	-4	GABA	14
GAL2 (h) (agonist radioligand)	-4	galanin	0.55
CXCR2 (IL-8B) (h) (agonist radioligand)	5	IL-8	0.058
CCR1 (h) (agonist radioligand)	-8	MIP-1alpha	0.042
H1 (h) (antagonist radioligand)	1	pyrilamine	1.5
H2 (h) (antagonist radioligand)	-16	cimetidine	510
MC4 (h) (agonist radioligand)	5	NDP-alpha -MSH	0.42
MT1 (ML1A) (h) (agonist radioligand)	5	melatonin	0.48
M1 (h) (antagonist radioligand)	-18	pirenzepine	18
M2 (h) (antagonist radioligand)	-3	methoctramine	29
M3 (h) (antagonist radioligand)	5	4-DAMP	1
NK2 (h) (agonist radioligand)	3	[Nleu10]-NKA (4-10)	1.5
NK3 (h) (antagonist radioligand)	-8	SB 222200	9.9
Y1 (h) (agonist radioligand)	-7	NPY	0.1
Y2 (h) (agonist radioligand)	-1	NPY	0.029
NTS1 (NT1) (h) (agonist radioligand)	-12	neurotensin	0.35
delta 2 (DOP) (h) (agonist radioligand)	2	DPDPE	1.5
kappa (KOP) (agonist radioligand)	-3	U 50488	0.64
mu (MOP) (h) (agonist radioligand)	-2	DAMGO	0.25

Assay	% Inhibition of reference (10 µM)	Reference Compound	K_i Ref (nM)
NOP (ORL1) (h) (agonist radioligand)	-5	nociceptin	0.11
EP4 (h) (agonist radioligand)	-15	PGE2	0.16
5-HT1A (h) (agonist radioligand)	-7	8-OH-DPAT	0.21
5-HT1B (antagonist radioligand)	-1	serotonin	6.9
5-HT2A (h) (antagonist radioligand)	-6	ketanserin	0.44
5-HT2B (h) (agonist radioligand)	17	(±)DOI	2.7
5-HT3 (h) (antagonist radioligand)	8	MDL 72222	4.8
5-HT5a (h) (agonist radioligand)	-24	serotonin	66
5-HT6 (h) (agonist radioligand)	-3	serotonin	72
5-HT7 (h) (agonist radioligand)	-10	serotonin	0.16
sst (non-selective) (agonist radioligand)	0	somatostatin-14	0.2
VPAC1 (VIP1) (h) (agonist radioligand)	-12	VIP	0.27
V1a (h) (agonist radioligand)	-4	[d(CH2)51,Tyr(Me)2]-AVP	1.2
Ca2+ channel (L, verapamil site) (phenylalkylamine) (antagonist radioligand)	-1	D 600	18
KV channel (antagonist radioligand)	-4	alpha -dendrotoxin	0.22
SKCa channel (antagonist radioligand)	5	apamin	0.0048
Na+ channel (site 2) (antagonist radioligand)	7	veratridine	7400
Cl- channel (GABA-gated) (antagonist radioligand)	-2	picrotoxinin	130
norepinephrine transporter (h) (antagonist radioligand)	-6	protriptyline	2.6
dopamine transporter (h) (antagonist radioligand)	13	BTCP	5.6
5-HT transporter (h) (antagonist radioligand)	-2	imipramine	1.1

Figure S1 related to Figure 2 Inhibition of KDM4/5 mediated H3K4me3 demethylation in cells

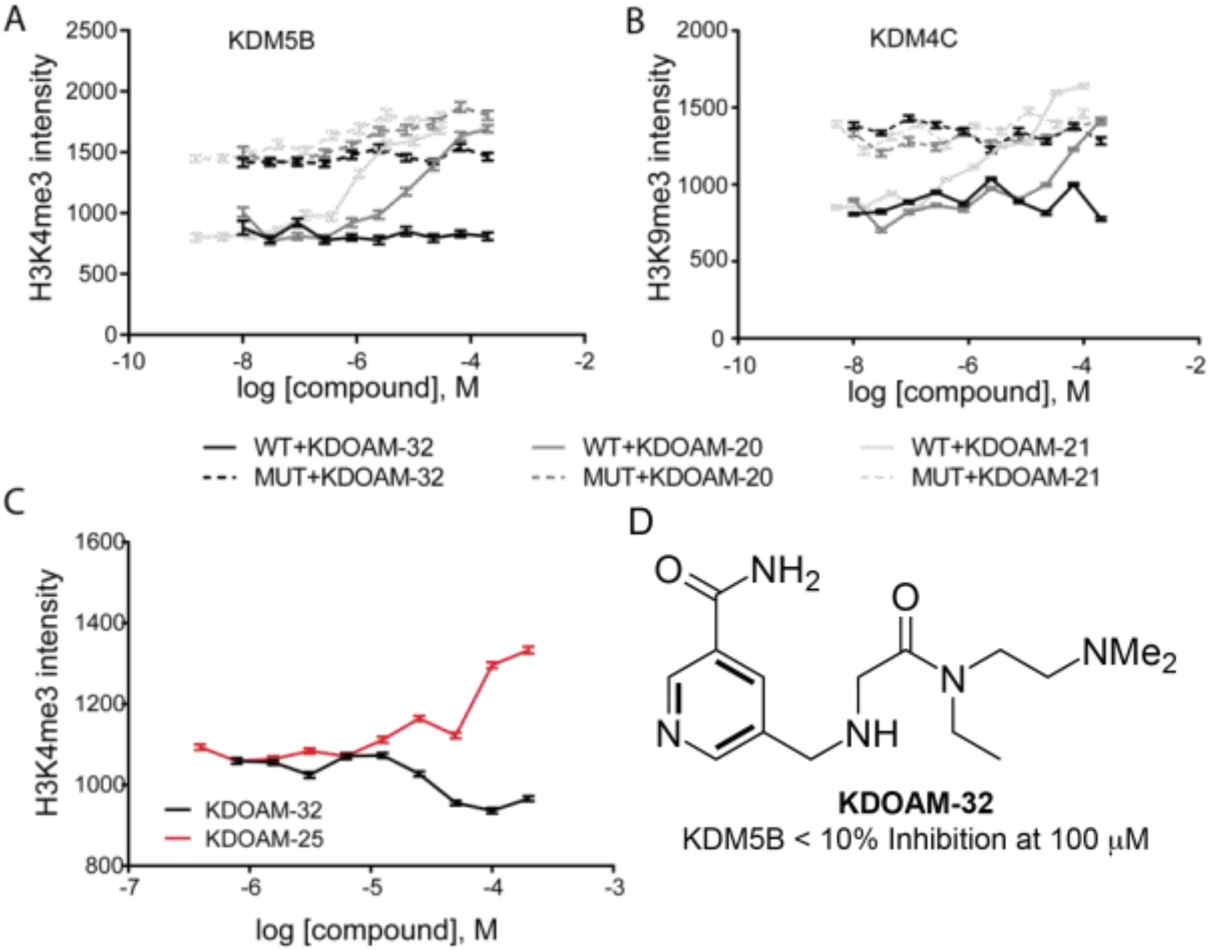

(A) EC_{50} curves of KDOAM-20, KDOAM-21 and KDOAM-32 in KDM5B and KDM4C (B) transfected HeLa cells (WT = wildtype; MUT = inactive mutant). (C) EC_{50} curves of KDOAM-25 and KDOAM-32 in untransfected HeLa cells. (D) KDOAM-32 is an inactive Control for KDOAM-25.

Figure S2 related to Figure 1 KDM5B Biochemical assay at different 2-OG concentrations

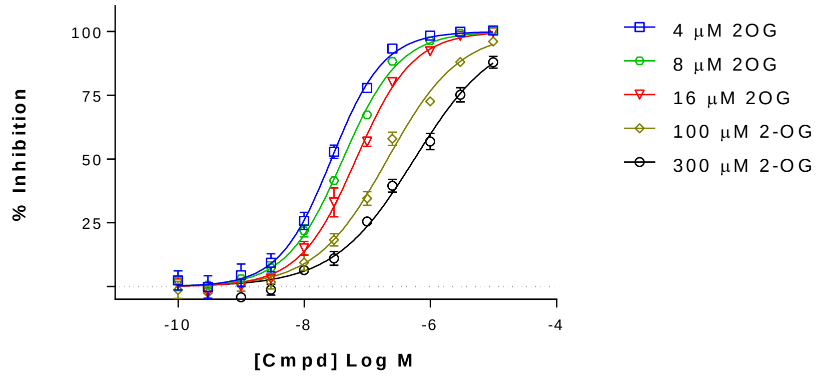

2OG Concentration	IC50 (μM)	K_i
4 μM 2OG	0.027	0.005
8 μM 2OG	0.041	0.005
16 μM 2OG	0.066	0.005
100 μM 2OG	0.210	0.003
300 μM 2OG	0.558	0.003

Figure S3 related to Figure 4 KDOAM-25 cytotoxicity

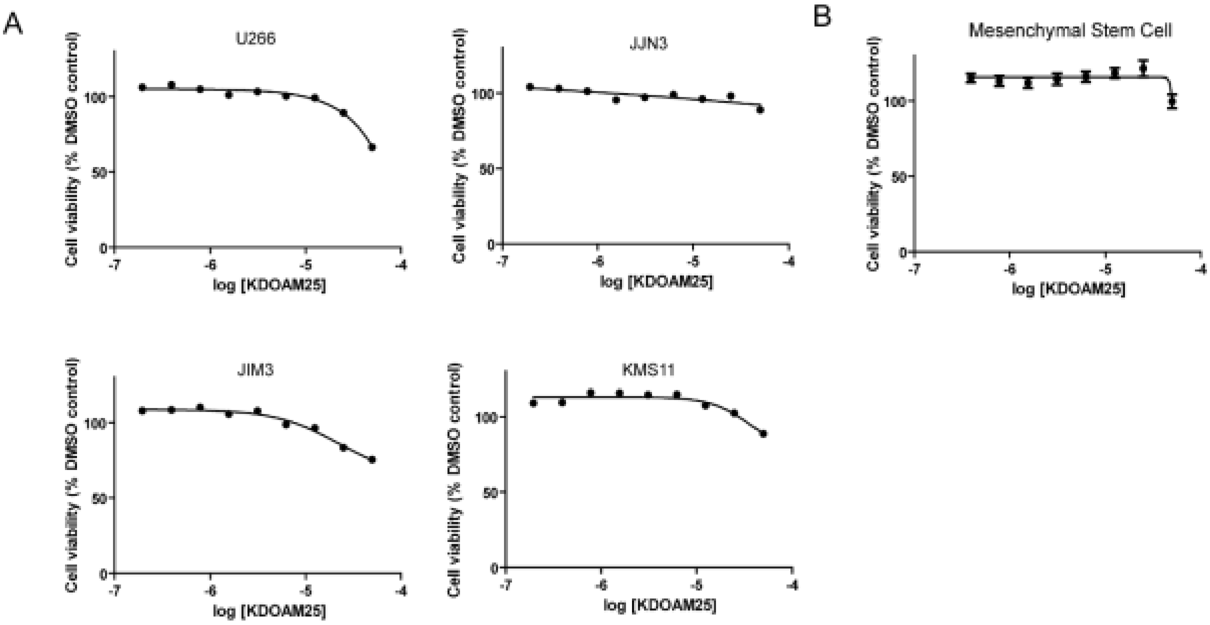

KDOAM-25 does not decrease cell viability in a range of multiple myeloma cell lines (A) or Mesenchymal Stem Cells (B) treated for 7 days. Data plotted represents the mean and standard deviation of four independent experiments.

Supplemental Experimental Procedures

General Experimental for Chemical Synthesis

Reactions were monitored using thin layer silica gel chromatography (TLC) 0.25 mm silica gel 60F plates from Merck. Plates were visualized by irradiation under UV lamp. Products were purified by flash chromatography on Biotage Isolera system using prepacked SNAP-Ultra, amino-functionalised or KP-Sil silica columns. Mass spectra were recorded using a Micromass Platform 1 spectrometer, operating in the positive or negative ion mode or HPLC-MS measurements were carried out using a Waters Autopurification System equipped with Waters 2489 UV/Vis detector, Waters 2424 ELS detector and SQ Detector 2. Chromolite Performance RP-18e, 3x100 mm and Phenomenex Kinetex 5μ EVO C18 100A, 3x100 mm columns were used for the analytical measurements using a gradient program; Chromolite SemiPrep RP18-e 10x100 mm and Phenomenex 5μ EVO C18 100A 21.2x150 mm columns were used for the preparative HPLC purifications using a gradient program; Eluent I: acetonitrile/water = 5/95 with 20 mM ammonium acetate buffer, pH 6.0, Eluent II: acetonitrile/water = 80/20 with 20 mM ammonium acetate buffer, pH 6.0. LCMS measurements were carried out using a WATERS sunfire C18 column using electrospray ionization, operating in the positive ion mode; separation was achieved using a linear gradient of solvent A (water + 0.01% CF_3CO_2H) and solvent B (acetonitrile + 0.01% CF_3CO_2H), eluting at a flow rate of 1 mL/min and monitoring at 254 nm: 0% B over 2 min, 0% B to 100% B over 16 min and 100% B over 2 min. NMR experiments were run on a Bruker Avance III 400 system (400.1 MHz for ^{1}H, 100.6 MHz for ^{13}C) equipped with a BBI probe and Z-gradients or a Bruker AV400 system (400.2 MHz for ^{1}H, 100.6 MHz for ^{13}C). Spectra were acquired at 300 K using deuterated dimethyl sulfoxide (d6-DMSO), deuterated chloroform ($CDCl_3$) or deuterated methanol (CD_3OD) as solvents and calibrated on the residual non-deuterated solvent signal. High resolution mass spectra (HRMS) were recorded using Bruker MicroTOF internally calibrated with polyalanine. Elemental analyses were recorded by the elemental analysis service of London Metropolitan University.

Synthesis of KDOAM-1

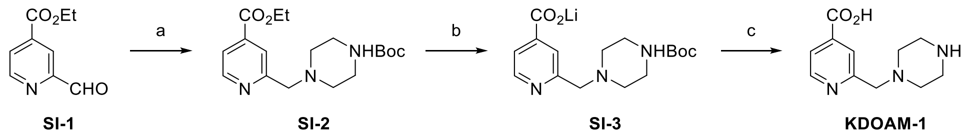

Reagents and conditions (a) *tert*-butyl piperazine-1-carboxylate, $NaBH(AcO)_3$, AcOH, CH_2Cl_2, quant.; (b) LiOH (aq), MeCN, quant.; (c) trifluoroacetic acid (TFA), 48%.

***tert*-Butyl 4-[(4-(Ethoxycarbonyl)pyridin-2-yl)methyl]piperazine-1-carboxylate (SI-2)**

tert-Butyl piperazine-1-carboxylate (1.15 g, 6.20 mmol) was added to a solution of ethyl 2-formylisonicotinate **SI-1** (Bavetsias et al., 2016) (735 mg, 4.10 mmol) in CH_2Cl_2 (20 mL) and the resulting solution stirred at room temperature for 1 h. Glacial acetic acid (3 drops) was added followed by $NaBH(AcO)_3$ (1.31 g, 6.20 mmol) and the resulting suspension stirred at room temperature for 16 h. The reaction mixture was diluted with water (20 mL), neutralised with saturated aq. sodium bicarbonate and extracted with CH_2Cl_2 (2 × 20 mL). The organic layers were washed with brine (20 mL) then combined, dried over magnesium sulfate and concentrated *in vacuo* to give **SI-2** as a colourless oil (1.44 g, quant.). R_f 0.30 (97.5:2.5 dichloromethane–methanol); LCMS (ES+): RT 9.6 min; *m/z* 350 $[M+H]^+$; ^{1}H-NMR (400 MHz, $CDCl_3$) 1.35–1.46 (12H, m), 2.42–2.48 (4H, m), 3.42–3.48 (4H,

m), 3.72 (2H, s), 4.39 (2H, q, *J* 7.0), 7.71 (1H, dd, *J* 5.0, 1.5), 7.92 (1H, app. s), 8.69 (1H, d, *J* 5.0); ^{13}C-NMR (125 MHz, $CDCl_3$) 14.1, 28.3, 42.8, 43.8, 52.9, 61.8, 64.1, 79.6, 121.4, 122.5, 138.2, 150.0, 154.7, 159.1, 165.2; HRMS: found 372.1891, calc. for $C_{18}H_{27}N_3NaO_4^+$ 372.1894.

Lithium 2-[(4-(*tert*-Butoxycarbonyl)piperazin-1-yl)methyl]isonicotinate (SI-3)

Lithium hydroxide (1 M, aq, 0.20 mL, 0.20 mmol) was added to a solution of *tert*-butyl 4-[(4-(ethoxycarbonyl)pyridin-2-yl)methyl]piperazine-1-carboxylate **SI-2** (70 mg, 0.20 mmol) in acetonitrile (1.0 mL) and water (0.8 mL) and the resulting solution stirred at room temperature for 16 h. The reaction mixture was concentrated *in vacuo* and the residue washed with cyclohexane (3 × 1 mL) to give **SI-3** as a yellow solid (68 mg, quant.). LCMS (ES+): RT 8.6 min (94%); *m/z* 322 $[M+H]^+$; ^{1}H-NMR (400 MHz, CD_3OD) 1.46 (9H, s), 2.45–2.52 (4H, m), 3.44–3.51 (4H, m), 3.71 (2H, s), 7.74 (1H, d, *J* 5.0), 7.97 (1H, s), 8.52 (1H, d, *J* 5.0); ^{13}C-NMR(100 MHz, CD_3OD) 28.8, 44.3, 45.3, 54.2, 64.9, 81.3, 123.5, 124.6, 148.5, 149.8, 156.5, 159.4, 172.6; HRMS: found 344.1577, calc. for $C_{16}H_{23}N_3NaO_4^+$ 344.1581

2-(Piperazin-1-ylmethyl)isonicotinic Acid Bis(trifluoroacetate) Monohydrate (KDOAM-1)

A solution of lithium 2-[(4-(*tert*-butoxycarbonyl)piperazin-1-yl)methyl]isonicotinate **SI-3** (61 mg, 0.19 mmol) in trifluoroacetic acid (1.0 mL) was stirred at room temperature for 16 h. The reaction mixture was concentrated *in vacuo* and the residue purified by strong-cation exchange chromatography, washing with methanol and eluting with methanolic ammonia (5% v/v solution of conc. ammonia in methanol) to give an off-white solid that was taken up in acetonitrile (1 mL). Trifluoroacetic acid (35 µL, 0.46 mmol) was added, the resulting suspension filtered and the filtrate concentrated *in vacuo*, azeotroping with water (2 × 2 mL) followed by ethyl acetate (2 × 2 mL) to give **KDOAM-1** as a beige gum (43 mg, 48%). LCMS (ES+): RT 1.9 min; *m/z* 222 $[M+H]^+$; ^{1}H-NMR (400 MHz, CD_3OD) 3.11–3.22 (4H, m), 3.39–3.48 (4H, m), 4.22 (2H, s), 7.98 (1H, d, *J* 5.0), 8.13 (1H, s), 8.79 (1H, d, *J* 5.0); ^{13}C-NMR (100 MHz, CD_3OD) 43.8, 50.7, 62.2, 124.7, 125.3, 142.7, 150.3, 156.4, 167.1; HRMS: found 222.1232, calc. for $C_{11}H_{16}N_3O_2^+$ 222.1237; elemental analysis: $C_{15}H_{19}F_6N_3O_7$ requires C, 38.55; H, 4.1; N, 9.0%; found C, 38.4; H, 3.9; N, 9.1%.

Synthesis of KDOAM-21

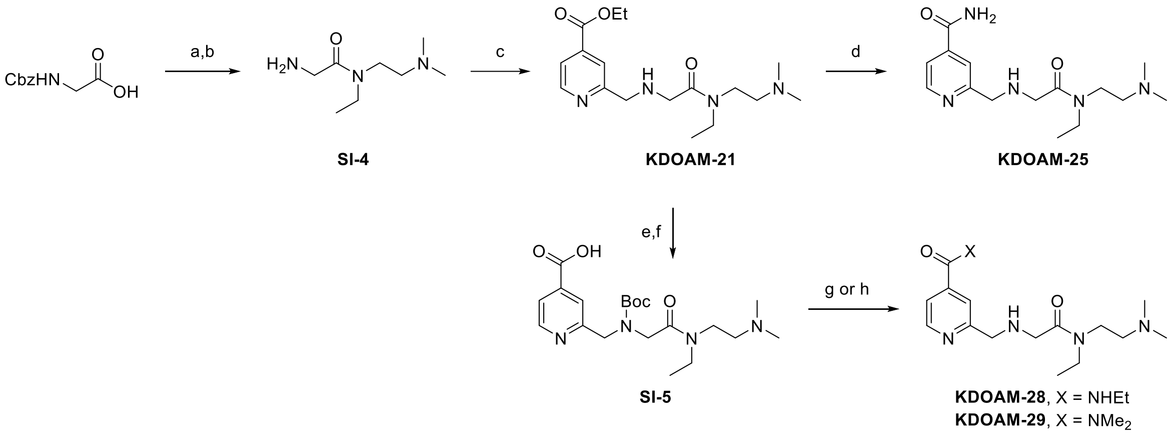

Reagents and conditions (a) *N*-ethyl-*N',N'*-dimethylethane-1,2-diamine, T3P, TEA, CH_2Cl_2, 64%; (b) cyclohexene, Pd/C, EtOH, 90%.; (c) **SI-1**, $NaBH(AcO)_3$, DCE, 75%; (d) NH_3, MeOH, 82%; (e) Boc_2O, TEA, DCE, 52%; (f) KOH, THF/MeOH/H_2O, 64%; (g) i. $EtNH_2$, HATU, DBU, CH_2Cl_2, 53%, ii. HCl, dioxane, 64%; (h) i. Me_2NH, HATU, DBU, CH_2Cl_2, 48%, ii. HCl, dioxane, 76%.

2-amino-*N*-(2-(dimethylamino)ethyl)-*N*-ethylacetamide (SI-4)

Step 1, benzyl (2-((2-(dimethylamino)ethyl)(ethyl)amino)-2-oxoethyl)carbamate: ((Benzyloxy)carbonyl)-glycine (6 g, 28.7 mmol) was dissolved in anhydrous CH_2Cl_2 (25 mL), then 2,4,6-tripropyl-1,3,5,2,4,6-trioxatriphosphinane 2,4,6-trioxide (T3P) (27.9 ml, 43.0 mmol) and triethylamine (TEA) (6.00 ml, 43.0 mmol) were sequentially added. After 10 minutes, *N*-ethyl-*N'*,*N'*-dimethylethane-1,2-diamine (4.52 ml, 28.7 mmol) was added. The solution was stirred at room temperature for 16 h, then diluted with CH_2Cl_2 and washed with 1N NaOH. The aqueous phase was extracted with additional CH_2Cl_2 (2 x 30 mL).The organic phase was dried over Na_2SO_4, filtered and concentrated to dryness. Product was purified on Biotage Isolera by silica chromatography with eluting system CH_2Cl_2/MeOH (from 100:0 to 80:20). (5.6 g, 64%) was recovered in pure form. LCMS (ES+): RT = 1.88min; *m/z* = 308 $[M-H]^+$; ^{1}H-NMR (d_6-DMSO): δ = 7.40-7.29 (m, 5H), 7.24 (t, 1H, *J* = 5.7 Hz), 5.04 (s, 2H), 3.86 (dd, 2H, *J* = 5.7, 15.0 Hz), 3.33-3.25 (m, 4H), 2.35 (m, 2H), 2.20-2.12 (m, 6H), 1.06 (m, 3H) ppm.

Step 2: Benzyl (2-((2-(dimethylamino)ethyl)(ethyl)amino)-2-oxoethyl)carbamate (6.6 g, 21.47 mmol) was dissolved in EtOH (350 mL), then cyclohexene (43.5 mL, 429 mmol) and palladium on activated charcoal (5.09 g, 4.29 mmol) were added in one portion. The solution was degassed (vacuum/N_2 cycles, 3 times) and stirred at 60 °C for 2 h. The solution was then filtered through a pad of Celite and the organic phase was concentrated to dryness. The product (5.7g, 90%) was pure enough for further elaborations. LCMS (ES+): RT = 0.34min; *m/z* = 174 $[M-H]^+$; ^{1}H-NMR (d_6-DMSO): δ = 5.06 (bs, 4H), 3.52-3.20 (m, 6H), 2.45-2.28 (m, 2H), 2.22-2.12 (m, 6H), 1.88 (s, 6H), 1.13-0.99 (m, 3H) ppm.

Ethyl 2-(((2-((2-(Dimethylamino)ethyl)(ethyl)amino)-2-oxoethyl)amino)methyl)isonicotinate (KDOAM-21)

A solution of ethyl 2-formylisonicotinate (**SI-1**) (0.69 g, 3.85 mmol) and 2-amino-*N*-(2-(dimethylamino)ethyl)-*N*-ethylacetamide (**SI-4**) (1.695 g, 5.78 mmol) in 1,2-dichloroethane (DCE) (80 mL) was stirred at room temperature for 15 min, then $NaBH(AcO)_3$ (1.306 g, 6.16 mmol) was added in one portion. The solution was stirred at room temperature for 16 h, then the solvent was evaporated. Purification on Biotage Isolera by RP silica chromatography with eluting system H_2O/MeCN from 95:5 to 0:100, afforded a first crop of product (0.900g, 51%) as double acetate salt, and a second crop of product (0.316g, 24%) as free base. LCMS (ES+): RT =1.04min; *m/z* = 337 $[M-H]^+$; Free base, mixture of rotamers: ^{1}H NMR (400 MHz, Methanol-d_4) δ 8.71 (dd, *J* = 5.1, 0.9 Hz, 1H), 8.01 (dd, *J* = 1.6, 0.8 Hz, 1H), 7.82 (dd, *J* = 5.1, 1.6 Hz, 1H), 4.44 (q, *J* = 7.1 Hz, 2H), 4.02 (s, 2H), 3.58 (s, 2H), 3.56 – 3.34 (m, 2H), 2.63 – 2.45 (m, 4H), 2.32 (d, *J* = 38.9 Hz, 6H), 1.42 (t, *J* = 7.1 Hz, 3H), 1.16 (dt, *J* = 21.6, 7.1 Hz, 3H); ^{13}C NMR (101 MHz, Methanol-d_4) δ 170.79, 164.87, 160.10, 149.57, 138.81, 121.47, 121.29, 61.61, 57.08, 56.07, 53.41, 48.60, 44.36, 44.12, 42.56, 41.58, 13.07, 12.65, 11.69.

Synthesis of KDOAM-25

2-(((2-((2-(Dimethylamino)ethyl)(ethyl)amino)-2-oxoethyl)amino)methyl)isonicotinamide (KDOAM-25)

The ethyl ester, **KDOAM-21** (0.3 g, 0.657 mmol), was dissolved in a solution of ammonia in methanol (7 M, 15.02 mL, 105 mmol) and the mixture was stirred at room temperature for 20h. After evaporation of the solvent, the crude was purified by Biotage Isolera automated chromatography (NH_2-Si cartridge, 11g) with eluting system CH_2Cl_2/MeOH from 100:0 to 95:5, affording pure product (0.165 g, 82%). The HCl salt was formed by dissolving the free base in a solution of HCl in dioxane (4M, 2 mL, 8 mmol) and the mixture was stirred at room

temperature for 6h. After evaporation of solvent and lyophilisation, the product (0.014 g, quant.) was recovered in pure form as an amorphous solid. LCMS (ES-/+): RT = 0.60 min; m/z = 306 $[M-H]^-$; m/z = 308 $[M-H]^+$; ^{1}H-NMR (CD_3OD): δ = 8.69 (dd, 1H, J = 0.5, 5.1 Hz), 7.77 (s, 1H), 7.71 (dd, 1H, J = 1.5, 5.1 Hz), 4.47 (s, 2H), 4.20 (s, 2H), 3.74 (t, 2H, J = 6.0 Hz), 3.35-3.26 (m, 4H), 2.88 (s, 6H), 1.16 (t, 3H, J = 7.1 Hz) ppm. ^{13}C-NMR (CD_3OD): δ = 167.7, 166.7, 151.9, 149.5, 143.2, 121.5, 120.9, 55.4, 50.0, 42.8, 41.5, 39.8, 12.1 ppm. HRMS: found 308.2078, calc. for $C_{15}H_{26}N_5O_2^+$ 308.2081.

Synthesis of KDOAM-28

2-(((*tert*-Butoxycarbonyl)(2-((2-(dimethylamino)ethyl)(ethyl)amino)-2-oxoethyl)amino)-methyl)iso-nicotinic acid (SI-5)

Step 1, Ethyl 2-(((*tert*-Butoxycarbonyl)(2-((2-(dimethylamino)ethyl)(ethyl)amino)-2-oxoethyl)amino)-methyl)isonicotinate: To a solution of **KDOAM-21** (0.45 g, 1.338 mmol) in CH_2Cl_2 (135 mL), di-tert-butyl dicarbonate (Boc_2O) (0.321 g, 1.471 mmol) and TEA (0.559 mL, 4.01 mmol) were sequentially added in one portion. The solution was stirred at room temperature for 1.5 h. After evaporation of the solvent, the crude product was purified by Biotage Isolera automated RP chromatography (C_{18} cartridge, 12 g) with eluting system H_2O/MeCN from 95:5 to 0:100, affording pure product (0.300 g, 51%). LCMS (ES+): RT = 2.25min; *m/z* = 437 $[M-H]^+$; ^{1}H-NMR (d_6-DMSO): δ = 8.73-8.70 (m, 1H), 7.81-7.70 (m, 2H), 4.57-4.47 (m, 2H), 4.41-4.32 (m, 2H), 4.22-4.04 (m, 2H), 3.30-3.19 (m, 4H), 2.38-2.30 (m, 2H), 2.21-2.05 (m, 6H), 1.41-1.23 (m, 12H), 1.14-0.97 (m, 3H) ppm.

Step 2: The product of the previous reaction (0.300 g, 0.687 mmol) in a 1:1:1 mixture of THF/MeOH/H_2O (24 mL) was treated with KOH (0.116 g, 2.06 mmol). The solution was stirred at room temperature for 16 h, then the solution was adjusted to pH 3 with 1N HCl. After evaporation of solvents, the crude product was purified by Biotage Isolera automated RP chromatography (C_{18} cartridge, 12 g) with eluting system H_2O/MeCN from 95:5 to 0:100, affording pure product (0.18 g, 64%) as the double hydrochloric salt. LCMS (ES+): RT = 0.62 min; *m/z* = 307 $[M-H-Boc]^-$; *m/z* = 309 $[M+H-Boc]^+$; ^{1}H-NMR (d_6-DMSO): δ = 8.56-8.31 (m, 1H), 7.73-7.41 (m, 2H), 4.50-4.38 (m, 2H), 4.22-4.03 (m, 2H), 3.63-3.55 (m, 2H), 3.39-3.25 (m, 2H), 2.97-2.86 (m, 2H), 2.62-2.54 (m, 6H), 1.41-1.19 (m, 9H), 1.18-0.99 (m, 3H) ppm.

2-(((2-((2-(Dimethylamino)ethyl)(ethyl)amino)-2-oxoethyl)amino)methyl)-*N*-ethylisonicotinamide (KDOAM-28)

To a solution of **SI-5***2HCl (20 mg, 0.042 mmol) and 1,8-diazabicycloundec-7-ene (DBU) (6.21 µL, 0.042 mmol) in CH_2Cl_2 (2mL), 1-[bis(dimethylamino)methylene]-1H-1,2,3-triazolo[4,5-b]pyridinium 3-oxide hexafluorophosphate (HATU) (47.4 mg, 0.125 mmol) was added in one portion. After 5 min of stirring, ethylamine (2 M solution in THF, 31 µL, 0.062 mmol) was added in one portion. The solution was stirred at room temperature for 20 h. After evaporation of the solvent, the crude product was purified by Biotage Isolera automated RP chromatography (C_{18} cartridge, 12g) with eluting system H_2O/MeCN from 95:5 to 0:100, affording pure product (0.013 g, 72%). LCMS (ES+): Rt = 1.92 min; *m/z* = 436 $[M-H]^+$.

To a solution of the purified product from the previous reaction (0.013 g, 0.030 mmol), hydrogen chloride in 1,4-dioxane (1.5 ml, 0.030 mmol) was added in one portion, followed by addition of water (0.5 mL) and a drop of 37% HCl solution. The reaction was stirred at room temperature for 3 h. After evaporation of the solvent, the crude product was purified by Biotage Isolera automated RP chromatography (C_{18} cartridge, 12g) with eluting

system H_2O/MeCN from 95:5 to 0:100, affording pure product (0.007 g, 53%) in pure form. LCMS (ES+): RT = 1.63min; *m/z* = 336 $[M-H]^+$; ^{1}H-NMR (CD_3OD): δ = 8.60-8.67 (m, 1H), 7.76 (bs, 1H), 7.69-7.66 (m, 1H), 4.48 (s, 2H), 4.21 (s, 2H), 3.77-3.72 (m, 2H), 3.38-3.28 (m, 6H), 2.89 (bs, 6H), 1.25-1.11 (m, 6H) ppm. ^{13}C-NMR (101 MHz, d_6-DMSO) δ 166.57, 164.36, 153.12, 149.96, 143.07, 134.68, 121.47, 53.92, 50.69, 47.59, 45.72, 42.86, 41.30, 34.67, 15.04, 13.70, 8.87.

Synthesis of KDOAM-29

To a solution of **SI-5** (30 mg, 0.062 mmol) and DBU (0.023 ml, 0.156 mmol) in CH_2Cl_2 (2mL), HATU (71 mg, 0.187 mmol) was added in one portion. After 5 minutes of stirring, dimethylamine*HCl (7.62 mg, 0.093 mmol) was added in one portion. The solution was stirred at room temperature for 2 0h. After evaporation of the solvent, the crude was purified by Biotage Isolera automated RP chromatography (C_{18} cartridge, 12 g) with eluting system H_2O/MeCN from 95:5 to 0:100, affording pure product (0.013g, 48%) in pure form. RT = 1.92 min; ESI-MS: *m/z* = 436 $[M-H]^+$. To a solution of the purified product from the previous reaction (30 mg, 0.059 mmol), hydrogen chloride in 1,4-dioxane (1.5 ml, 0.030 mmol) was added in one portion, followed by addition of water (0.5 mL) and a drop of 37% HCl solution. The reaction was stirred at room temperature for 3 h. After evaporation of the solvent, the crude was purified by Biotage Isolera automated RP chromatography (C_{18} cartridge, 12 g) with eluting system H_2O/MeCN from 95:5 to 0:100, affording pure product (0.02g, 76%) in pure form. LCMS (ES^+): RT = 0.28 min; *m/z* = 336 $[M-H]^+$; ^{1}H-NMR (CD_3OD): δ = 8.76 (dd, 1H, *J* = 0.5, 5.0 Hz), 7.52 (m, 1H), 7.47 (dd, 1H, *J* = 1.3, 5.0 Hz), 4.56 (s, 2H), 4.32 (s, 2H), 3.85 (t, 2H, *J* = 6.0 Hz), 3.46-3.38 (m, 4H), 3.15 (s, 3H), 3.00 (s, 9H), 1.28 (t, 3H, *J* = 6.0 Hz) ppm; ^{13}C-NMR (CD_3OD): δ = 167.7, 166.7, 151.9, 149.7, 143.2, 121.1, 120.1, 55.3, 50.1, 42.8, 41.5, 39.9, 38.2, 34.0, 12.1.

Synthesis of KDOAM-32

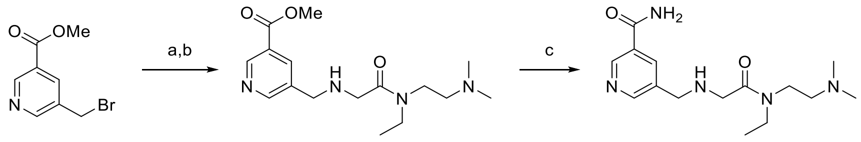

Reagents and conditions (a) $AgBF_4$, TEA, DMSO, 41%; (b) **SI-4**, $NaBH(AcO)_3$, DCE, 41%; (c) NH_3, MeOH, 66%.

Methyl 5-(((2-((2-(Dimethylamino)ethyl)(ethyl)amino)-2-oxoethyl)amino)methyl)nicotinate (SI-6)

Step 1: To a solution of methyl 5-(bromomethyl)nicotinate (200 mg, 0.869 mmol) in DMSO (5 mL) was added a solution of silver(I) tetrafluroborate (220 mg, 1.130 mmol) in dry DMSO (1 mL) . The suspension was stirred 40 min at RT, then TEA (0.364 mL, 2.61 mmol) was added and the mixture was stirred for further 15 min. The reaction was diluted with EtOAc and the organic solution was washed with sat. $NaHCO_3$ and brine. The aq. layer was further extracted with CH_2Cl_2 (2 x 20 mL) and EtOAc (2 x 20 mL). The combined organic fractions were dried (Na_2SO_4) and concentrated. The crude residue residue was purified by flash chromatography eluting with a gradient of 0 to 60% EtOAc in Cyclohexane. Methyl 5-formylnicotinate was obtained as white fluffy solid (60 mg, 41%). LCMS (ES+): RT 1.04 min; *m/z* 165 $[M+H]^{+.}$ ^{1}H NMR (400 MHz, $CDCl_3$) δ 10.21 (s, 1H), 9.45 (d, *J* = 2.1 Hz, 1H), 9.27 (d, *J* = 2.1 Hz, 1H), 8.77 (t, *J* = 2.1 Hz, 1H), 4.03 (s, 3H).

Step 2: $NaBH(AcO)_3$ (90 mg, 0.424 mmol) was added to a solution of 2-amino-*N*-(2-(dimethylamino)ethyl)-*N*-ethylacetamide (**SI-4**) (118 mg, 0.678 mmol) and methyl 5-

formylnicotinate (70 mg, 0.424 mmol) in DCE (5 mL). The reaction was stirred for 3 h at RT. The solvent was removed under reduced pressure and the crude residue was purified by flash chromatography eluting with a gradient of 0 to 10% (MeOH + 1% NH_3). The title compound was isolated as colourless oil (56 mg, 41%). LCMS (ES+): RT 0.73, *m/z* 322 [M+H]+. ^{1}H-NMR (400 MHz, $CDCl_3$) δ 9.09 (d, J = 2.1 Hz, 2H), 8.73 (d, J = 2.2 Hz, 2H), 8.30 (t, J = 2.2 Hz, 2H), 3.98 – 3.84 (m, 7H), 3.51 (t, J = 7.1 Hz, 2H), 3.47 – 3.30 (m, 4H), 3.35 – 3.19 (m, 2H), 2.53 (t, J = 7.1 Hz, 2H), 2.38 – 2.16 (m, 6H), 1.13 (td, J = 7.1, 5.3 Hz, 4H).

5-(((2-((2-(Dimethylamino)ethyl)(ethyl)amino)-2-oxoethyl)amino)methyl)nicotinamide (KDOAM-32)

Ammonia (4.96 mL, 34.7 mmol) in Methanol was added to a sealed flask containing methyl 5-(((2-((2-(dimethylamino)ethyl)(ethyl)amino)-2-oxoethyl)amino)methyl)nicotinate (**SI-6**) (56 mg, 0.174 mmol). The slightly yellow solution was stirred at RT for 12 h until complete conversion was observed by LCMS. The solvents were removed under reduced pressure and the crude residue was dissolved in CH_2Cl_2 and purified by flash chromatography on a 11 g KP-NH column, eluting with a gradient of 0 to 10% MeOH in DCM. The desired product was isolated as colourless oil (35 mg, 66%). LCMS (ES+): RT 0.36, *m/z* 308 $[M+H]^+$, ^{1}H-NMR (400 MHz, $CDCl_3$) δ 8.91 (d, *J* = 2.2 Hz, 1H), 8.61 (d, *J* = 2.1 Hz, 1H), 8.15 (d, *J* = 2.2 Hz, 1H), 6.86 (s, 1H), 6.35 (s, 1H), 3.81 (s, 2H), 3.44 – 3.28 (m, 4H), 3.24 – 3.13 (m, 2H), 2.38 (d, *J* = 7.1 Hz, 1H), 2.32 (s, 3H), 2.19 (s, 3H), 2.14 (s, 3H), 1.06 (q, *J* = 7.2 Hz, 3H). ^{13}C-NMR (101 MHz, $CDCl_3$) δ 170.30, 167.75, 152.36, 147.76, 135.31, 129.01, 58.04, 56.95, 51.49, 49.37, 45.59, 43.47, 41.82, 41.38, 13.97, 12.94.

Stability of KDOAM-21 and KDOAM-25 in DPBS

HPLC-MS measurements were carried out using a Waters Autopurification System equipped with Waters 2998 photodiode array detector, Waters 2424 ELS detector and ACQUITY QDa Detector. A Phenomenex 5µ Gemini-NX C18 110A, 4.6 x 250 mm column was used for the analytical measurements using a gradient program; Eluent A: acetonitrile/water = 5/95 with 20 mM ammonium acetate buffer, pH 6.0, Eluent B: acetonitrile/water = 80/20 with 20 mM ammonium acetate buffer, pH 6.0, eluting at a flow rate of 2 mL/min and monitoring at 254 nm: 0% B over 2 min, 0% B to 95% B over 2.5 min and 95% B over 2 min.

1 mM solutions of KDOAM-21 and KDOAM-25 in Dulbecco's Phosphate Buffered Saline (Sigma D8537) were prepared from 50 mM DMSO stocks of KDOAM-21 and KDOAM-25. The resulting solutions were maintained at 37 C and aliquots removed for analysis by HPLC-MS at 0 min, 20 min, 1 hour, 4 hours and 24 hours. The stability of KDOAM-21 and KDOAM-25 to decomposition to KDOAM-20 was determined by comparison of the integrated area of the peaks at 254 nm.

References

Bankevich, A., Nurk, S., Antipov, D., Gurevich, A.A., Dvorkin, M., Kulikov, A.S., Lesin, V.M., Nikolenko, S.I., Pham, S., Prjibelski, A.D., *et al.* (2012). SPAdes: a new genome assembly algorithm and its applications to single-cell sequencing. J Comput Biol *19*, 455-477.

Bavetsias, V., Lanigan, R.M., Ruda, G.F., Atrash, B., McLaughlin, M.G., Tumber, A., Mok, N.Y., Le Bihan, Y.-V., Dempster, S., Boxall, K.J., *et al.* (2016). 8-Substituted Pyrido[3,4-d]pyrimidin-4(3H)-one Derivatives As Potent, Cell Permeable, KDM4 (JMJD2) and KDM5 (JARID1) Histone Lysine Demethylase Inhibitors. J Med Chem *59*, 1388-1409.

Hopkinson, R.J., Tumber, A., Yapp, C., Chowdhury, R., Aik, W., Che, K.H., Li, X.S., Kristensen, J.B.L., King, O.N.F., Chan, M.C., *et al.* (2013). 5-Carboxy-8-hydroxyquinoline is a broad spectrum 2-oxoglutarate oxygenase inhibitor which causes iron translocation. Chem Sci *4*, 3110-3117.

Johansson, C., Velupillai, S., Tumber, A., Szykowska, A., Hookway, E., Nowak, R.P., Strain-Damerell, C., Gileadi, C., Philpott, M., Burgess-Brown, N., *et al.* (2016). Novel inhibitor chemotypes for human JARID1B/KDM5B enzymes highlight essential structure-activity relationships with KDM4 and KDM6-type histone demethylases. Nat Chem Biol.

Lambert, J.P., Tucholska, M., Pawson, T., and Gingras, A.C. (2014). Incorporating DNA shearing in standard affinity purification allows simultaneous identification of both soluble and chromatin-bound interaction partners. J Proteomics *100*, 55-59.

Schulz, M.H., Weese, D., Holtgrewe, M., Dimitrova, V., Niu, S., Reinert, K., and Richard, H. (2014). Fiona: a parallel and automatic strategy for read error correction. Bioinformatics (Oxford, England) *30*, i356-363.
